# Geographic provenance and environmental growing conditions as factors influencing phytochemical composition of Arabica green coffee beans

**DOI:** 10.1111/plb.70136

**Published:** 2025-11-11

**Authors:** I. Pettazzoni, G. Benati, S. Monari, E. De Angelis, L. Navarini, M. Ferri, A. Tassoni

**Affiliations:** ^1^ Department of Biological, Geological, and Environmental Sciences University of Bologna Bologna Italy; ^2^ Aromalab Illycaffè S.p.A., Area Science Park Trieste Italy; ^3^ Illycaffè S.p.A. Trieste Italy

**Keywords:** Arabica coffee beans, biogenic amines, caffeine, chlorogenic acids, polyphenols

## Abstract

- This study explores how bioactive compounds in green coffee beans (*Coffea arabica* L.) vary across different geographic regions, addressing the key question of how environmental factors shape coffee biochemistry and adaptation mechanisms to diverse conditions. Identifying these variations provides insight into how environmental and processing factors influence coffee's sensory quality.
- Samples from six major coffee‐producing regions were analysed for key bioactive compounds, including biogenic amines, caffeine, trigonelline, sucrose, free amino acids, and phenolics. Total polyphenol content and polyamine concentrations were measured, and PCA was used to differentiate samples based on chemical composition. A correlation analysis was specifically conducted for Brazilian samples, using meteorological and environmental data.
- Total polyphenol content ranged from 44.8 to 70.7 mg GAeq g^−1^ FW, with Brazilian samples having the highest levels. Putrescine, the most abundant polyamine, varied significantly (0.02–1.9 μg g^−1^ FW). PCA highlighted Ethiopian samples with high sucrose and low caffeine. Brazilian samples showed distinct separation based on key compounds, including putrescine, trigonelline, and amino acids. Environmental factors in Brazil correlated with polyamine and amino acid composition, suggesting associations with heat and drought tolerance.
- Environmental factors, particularly heat and drought, influence the biochemical profile of coffee beans. Polyamine levels correlate with stress tolerance, while amino acid composition reflects adaptations for osmotic protection. These findings enhance our understanding of coffee's biochemical adaptation to diverse climates and offer valuable insights for optimizing cultivation strategies in the face of climate change.

## Graphical abstract

Growth environment strongly influences green coffee bean biochemistry, linking phytochemical composition to adaptation under abiotic stress.

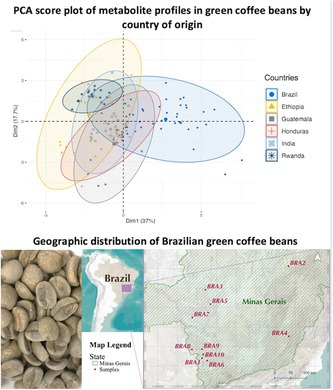

## INTRODUCTION

The coffee plant (*Coffea* spp., Rubiaceae) includes two commercially important species, *Coffea arabica* L. and *Coffea canephora* Pierre ex A. Froehner, commercially known, respectively, as Arabica and Robusta (Melese & Kolech 2021). Originating from Africa, coffee was first cultivated in Ethiopia for its favourable environmental conditions (Brücher 1977). Today, coffee is grown in multiple tropical and subtropical regions worldwide, with Brazil being the largest producer, accounting for 3.4 Mt in 2023 (FAOSTAT 2023). Arabica species thrives under specific climate conditions: consistent rainfall (1200–2200 mm year^−1^), moderate temperatures (15–24°C), well‐drained soil, moderate‐high altitude (950–1950 m), and shaded environments. The Robusta plant benefits from similar conditions: consistent rainfall (2200–3000 mm year^−1^), warm temperatures (18–36°C), well‐drained soil, and moderate altitudes (250–1500 m) (Oestreich‐Janzen 2013).

Coffee plant growth and post‐harvest production involve many stages, each of them impacting on the final quality of the beans, including both the presence of desirable compounds as precursors of aroma and taste, and the absence of defects that could negatively affect the sensory, physical and chemical properties (Halagarda & Obrok 2023). The flavour profile is deeply influenced by phytochemicals present in the beans, which contribute to key sensory attributes like acidity, bitterness, aroma, and body. These compounds undergo significant changes throughout the flowering, fruit development, and maturation, and their composition is heavily shaped by the plant's growing environment (De Castro & Marraccini 2006; Cheng *et al*. 2016). Factors such as temperature and rainfall can influence the uniformity of bean ripening by affecting the flowering period (de Oliveira Aparecido *et al*. 2018; López *et al*. 2021). Inconsistent flowering can lead to uneven maturation, producing beans with different concentrations and balance of these phytochemicals, responsible for the bean bitterness, acidity, and antioxidant properties (Oestreich‐Janzen 2013; López *et al*. 2021).

Variations in the flavour precursors can also occur following post‐harvest treatment through either dry or wet processing methods (Dias *et al*. 2012; Oestreich‐Janzen 2013). The dry processing method, typically used for Robusta, involves drying the entire coffee cherry. In contrast, the wet method, commonly applied to Arabica species, separates the pulp from the seed before drying through mechanical de‐pulping and fermentation (Halagarda & Obrok 2023). Ultimately, the relationship between genetic factors, environmental conditions, and processing methods is crucial in determining the unique chemical composition and flavour profile of the coffee bean.

Among the different compounds present in coffee beans, phenols and alkaloids are the most abundant secondary metabolites, and their concentrations are influenced by environmental and growing conditions. These compounds are produced by the plant to serve various roles, including defence molecules against different types of stresses (Grohar *et al*. 2021). Chlorogenic acids, which are esters of quinic acid, and hydroxylated *trans*‐cinnamic acids, such as *p*‐coumaric acid and caffeic acid, represent a significant portion of phenols in green coffee beans (Liczbiński & Bukowska 2022). In green beans of *C. arabica*, the predominant isoform of chlorogenic acid is 5‐O‐caffeoylquinic acid (5‐CQA), while other isomers, such as 3‐O‐caffeoylquinic acid (3‐CQA, neochlorogenic acid) and 4‐O‐caffeoylquinic acid (4‐CQA, cryptochlorogenic acid) are also present (Naveed *et al*. 2018). Another *trans*‐cinnamic acid derivative found in coffee beans is ferulic acid (FERA) (Farah & Donangelo 2006). These compounds are involved in determining coffee quality, specifically as precursors of desirable sensory attributes, as after roasting, some of them undergo hydrolysis, degradation, or isomerization influencing both taste and overall flavour (Jeszka‐Skowron *et al*. 2016). The chlorogenic acids overall composition and concentration vary according to species, maturation degree, agricultural practices, and environmental factors (Farah & Donangelo 2006; Sualeh *et al*. 2020).

Caffeine (CAF) is a purine alkaloid present in all aerial parts of the plant and synthesized as a chemical defence against herbivores and microbial pathogens. Its content is higher in young leaves and lower in aged leaves (Cheng *et al*. 2016). CAF is also formed in the pericarp of immature coffee fruits and gradually accumulates in the endosperm during bean development. CAF content is primarily genetically determined, varying among species and varieties, with Arabica coffee known for its lower amount compared to Robusta (Oestreich‐Janzen 2013; Cheng *et al*. 2016). Research also suggests that CAF biosynthesis is influenced by the plant growth environmental conditions and cultivation practices, such as temperature, light, and soil water content. It was suggested that warmer temperatures and higher light exposure significantly influence CAF biosynthesis in *C. arabica* leaves, particularly by enhancing the methylation process that converts theobromine to caffeine during the final step of CAF biosynthesis (Frischknecht *et al*. 1982). Similarly, in the pericarp tissues light strongly promotes this methylation stage of caffeine production (Oestreich‐Janzen 2013).

Trigonelline (TRI), a pyridine alkaloid derived from nicotinic acid, is the second most abundant alkaloid in coffee (Koshiro *et al*. 2006). Its content is higher in Arabica than in Robusta beans. TRI accumulates primarily in young stems and leaves, reaching highest concentrations before full ripeness. In mature coffee fruits, 70%–90% of TRI is localized in the seeds, with the remainder in the pericarp. Its biosynthesis follows a pattern similar to CAF, increasing in young seeds and declining in mature tissues (Koshiro *et al*. 2006; Ashihara 2015).

Together with CQA isomers and TRI, sucrose plays a crucial role in defining coffee quality and flavour, as non‐volatile compound serving as key precursor for volatile compounds that develop during roasting (Campa *et al*. 2004). Arabica coffee generally contains 30% more sucrose than Robusta, contributing to its superior sensory quality. During roasting, sucrose acts as a precursor in the Maillard reaction and caramelization leading to the formation of compounds such as furans, pyrazines, alkyl‐pyridines and pyrroles which contribute to coffee aroma (Ky *et al*. 2001).

Beyond species, factors such as variety, maturity, agricultural practices, and environmental conditions, particularly air temperature, light and water availability, can influence sucrose accumulation (Silva *et al*. 2005; Geromel *et al*. 2008; Stredansky *et al*. 2018).

Amino acids are fundamental building blocks for protein synthesis, involved in various physiological processes, including plant growth, nitrogen uptake and translocation, and seed development (Yang *et al*. 2020). The pool of free amino acids also plays a crucial role in the plant's response to environmental stresses by acting as osmoprotectants, antioxidants, and signalling molecules (Ghosh *et al*. 2021). Proline, for example, accumulates under drought, salinity, and heat stress, helping to maintain water content and to protect cells from damage (Suprasanna *et al*. 2014; Singh *et al*. 2022). Glutamate and γ‐aminobutyric acid (GABA), contribute to stress signalling and metabolic adjustments, while branched‐chain and aromatic amino acids are involved in drought resistance and pathogen defence (Ghosh *et al*. 2021; Singh *et al*. 2022). Besides being affected by environmental stresses (Joët, Laffargue, *et al*. 2010), the amino acid content can also be influenced by post‐harvest processes (Figueroa Campos *et al*. 2022). The levels of free amino acids consistently vary between the two species, with Robusta containing more than Arabica (Arnold *et al*. 1994).

Biogenic amines (BAs) are a family of low molecular weight nitrogenous compounds typically characterized by the presence of amino groups (−NH_2_) attached to an aliphatic or aromatic structure (Groppa & Benavides 2008; Chen *et al*. 2019). Depending on the number of alkyl groups bound to nitrogen atoms, amines are classified as primary, secondary, or tertiary. BAs can be present in free form, soluble conjugates, and insoluble conjugates (Bagni & Tassoni 2001; Bagni *et al*. 2006). The most common polyamines (PAs) are putrescine (PUT), spermidine (SPD), and spermine (SPM). Their role is central in regulating numerous biochemical and physiological functions (Chen *et al*. 2019). PUT, SPD, and SPM also play a crucial role in plant responses to abiotic stresses such as drought, salinity, extreme temperatures, oxidative stress as well as less‐studied stressors like mineral deficiencies, chilling, and wounding (Bagni *et al*. 2006; Groppa & Benavides 2008; Gill & Tuteja 2010; Chen *et al*. 2019). Among the stress‐induced changes, the rise in PUT and SPD is often linked to increased arginine decarboxylase isoforms (ADC1 and ADC2) and S‐adenosylmethionine decarboxylase (SAMDC) gene expression and activities (Bagni *et al*. 2006; Liu *et al*. 2015; Chen *et al*. 2019). Despite strong evidence of PAs involvement in stress tolerance, the mechanisms by which they vary under environmental plant growth conditions still require further research.

This study aims to examine the variation of different biochemical compounds, such as phenols, caffeine, sucrose, amino acids, and biogenic amines, in green coffee beans harvested from different countries, to better understand how climatic and environmental conditions influence coffee plant development and the accumulation of compounds that determine coffee flavour. Specifically, for Brazilian samples, where the exact cultivation coordinates are known, we seek to integrate biochemical analysis with environmental data to provide new insights that can help optimize cultivation strategies, improve stress resilience, and enhance both the sensory and nutritional properties of coffee. Ultimately, this research can assist farmers and producers in adapting to changing environmental conditions while maintaining or even improving coffee biochemical and sensory quality.

## MATERIAL AND METHODS

### Sample material and preparation

A total of 30 green *C. arabica* L. bean samples (wet‐processed) were provided (February–March 2022) by *illycaffè* S.p.A. (Trieste, Italy) after sourcing from separate geographic regions. Ten samples were collected from different locations of Minas Gerais (Brazil) (Table S1), while four samples each were sourced from associations of small local producers in Guatemala, Honduras, Ethiopia, Rwanda, and India, of which the exact growing location details are not available.

The commercial samples of green coffee beans were selected at the *illycaffè* S.p.A. company's facilities based on standard sorting procedures and considering several parameters, including colour, size, and moisture content (<11% w/w). The samples were stored at −20°C before being ground with an analytical mill (A 11 basic, IKA, Staufen im Breisgau, Germany). The ground seed powders were also stored at −20°C until the following analyses.

### Phenolic acid and alkaloid quantifications

Water extractions were carried out with boiling water, allowing high solubility of the target analytes. For each extraction 0.3 g FW seed powder was combined with 30 mL 100°C deionized water (1:100 S/L ratio) followed by incubation at room temperature (RT) for 10 min at 120 rpm rotatory shaking. The supernatants and pellets were separated by centrifugation (10 min, 5000 rpm, RT) and stored at −20°C until analysis.

Total polyphenols in aqueous extracts were measured with the Folin‐Ciocalteau assay (Ferri *et al*. 2013). Results were expressed as mg gallic acid (GA) equivalents gFW of coffee seeds (mg GAeq g^−1^ FW) based on a dose–response calibration curve (0–15 μg GA) measured at an absorbance of 765 nm using a microplate reader (VersaMax, Molecular Devices, San Jose, CA, USA).

Chlorogenic acid isomers (i.e., 5‐O‐caffeoylquinic acid (5‐CQA), 3‐O‐caffeoylquinic acid (3‐CQA), 4‐O‐caffeoylquinic acid (4‐CQA)), ferulic acid (FERA), caffeine (CAF) and trigonelline (TRI) content in aqueous extracts were quantified by HPLC separation, coupled with a diode array detector (DAD) (Ferri *et al*. 2009). Both standards and samples were diluted with milli‐Q water (1:5) and filtered (Phenex RC Membrane 0.2 μm, Phenomenex, Castel Maggiore, Bologna, Italy) before being injected into the HPLC‐DAD system (JASCO, Groß‐Umstadt, Germany; diode array detector PDA MD‐2010; column Gemini C18 110 Å, 5 μm, 4.6 mm × 250 mm, pre‐column SecurityGuard Ea, Phenomenex, Italy). The detection wavelengths were 270 nm for caffeine and trigonelline, and 323 nm for chlorogenic and ferulic acids. Two different standard concentrations, chosen to closely match the concentration range of the target analytes in the samples and injected in at least 10 technical repetitions along different days of analysis, were employed for caffeine, trigonelline and CQAs quantification.

### Sucrose quantification

For sucrose quantification, 1 g seed powder was added to 25 mL boiling Milli‐Q water and homogenized in an orbital shaker (Heidolph Unimax 1010, Schwabach, Germany) for 90 min at 24°C. Samples were centrifuged (8 min, 7000 rpm, RT) and supernatants filtered through a 0.45 μm nylon membrane filter (Phenomenex, Torrance, CA, USA). Sucrose analyses were carried out using an Agilent 1260 series HPLC system equipped with a refraction index detector (RID) (Agilent Technologies, Santa Clara, CA, USA) (Kulapichitr *et al*. 2019). The separation was carried out on a ZORBAX‐Carbohydrate analytical column (5 μm, 4.6 mm × 250 mm; Agilent Technologies). Elution was performed at 30°C with acetonitrile:water (60:40, v/v) as mobile phase (flow rate: 1 mL min^−1^). Standard stock solution was prepared by dissolving sucrose in Milli‐Q water (5.0 g L^−1^) then diluted to obtain the final calibration solutions of 5, 2.5, 1, 0.5, 0.25 g L^−1^. The standard stock solution was stored at 4°C and brought to 24°C before use.

### Free amino acid quantification

Free amino acid content was determined by HPLC separation coupled with a fluorometer detector (FLD) after sample derivatization with the AccQ‐Tag kit (Waters, Milford, CT, USA) following the manufacturer's instructions (Monari *et al*. 2019). The Amino Acid Hydrolysate Standard mixture (Waters) containing 2.5 mM of each amino acid (asp, ser, glu, gly, his, arg, thr, ala, pro, tyr, val, met, lys, ile, leu, phe), with the exception of cys (1.25 mM), was subjected to the same procedure.

Calibration with a single‐point concentration of the standard mix (at least 10 technical repetitions on different days of analysis) was employed for the quantification. After being filtered (Phenex RC Membrane 0.2 μm; Phenomenex, Italy), the derivatized samples were injected into an AccQ‐Tag Amino Acids C18 Column (4 μm, 3.9 mm × 150 mm; Waters). The detector was set to an excitation wavelength of 250 nm and emission wavelength of 395 nm. Elution was performed with a multi‐step linear gradient (flow rate: 1 mL min^−1^) (Monari *et al*. 2019).

### Biogenic amine quantification

Free biogenic amines determination was performed as in Tassoni *et al*. (2000). Ground seed powders (0.2 g FW) were extracted in 1.8 mL 4% (v/v) perchloric acid (PCA) and centrifuged (30 min, 10,000 rpm, 4°C). Three aliquots of supernatant (0.2 mL) were derivatized with dansyl‐chloride, toluene‐extracted and analysed by HPLC‐FLD (JASCO; Jasco 821‐FP in‐line spectrofluorometer detector) using a reverse‐phase C18 column (Gemini C18 110 Å, 5 μm, 4.6 mm × 250 mm; Phenomenex, Italy). Elution was performed with a multi‐step linear gradient (flow rate: 1 mL min^−1^) (Tassoni *et al*. 2000). Standard biogenic amines mixtures (1,3‐diaminopropane, cadaverine, putrescine, spermidine, spermine, tryptamine, phenylethylamine, histamine, and tyramine) were subjected to the same procedure. After each HPLC analysis, the injected compounds were identified and quantified using the ChromNAV CFR software (Jasco, Tokyo, Japan).

### Environmental data acquisition

The exact cultivation sites of Brazilian coffee samples, all in Minas Gerais state, were used to collect datasets of meteorological and environmental data for correlation analysis (Tables S1 and S2) from the Visual Crossing Corporation (2024) database, a global weather data service providing historical and real‐time meteorological data. Precise growing locations for the samples from Guatemala, Ethiopia, Honduras, Rwanda, and India were not available, as the suppliers in those countries are usually farming consortia that collect coffee from small local producers. The datasets include hourly and daily records from April to June 2021 (coffee bean maturation period estimated on the basis of harvesting period June–August 2021) of key parameters for each coffee bean collection location. For the target study period, mean values were calculated for all the variables of each location: elev (altitude above sea level in m), maximum, minimum and mean air temperatures recorded 2 m above the ground (Tmax, Tmin, Tmean in °C), relative humidity (Rh in %), cloud cover (Cc in %), soil temperature at 7, 28, 100, 255 cm depth (sT7, sT28, sT100, sT255 in °C), soil moisture at 7, 28, 100, 255 cm depth (sm7, sm28, sm100, sm255 in m^3^ m^−3^), average wind speed and maximum wind gusts recorded at a height of 10 m above the ground (wnd_spd, wnd_gst in km h^−1^), rainfall (precip in mm), daylight duration (day_dur, in sec), sunshine duration (sun_dur in sec), shortwave radiation (sw_rad in MJ m^−2^) and evapotranspiration (evap in mm) (Tables S1 and S2). The datasets were standardized for further statistical analysis by centering (subtracting the mean) and scaling (dividing by the standard deviation) each variable, making all variables contribute equally to the analysis (R Core Team 2023).

### Statistical analysis

All analyses were performed on three biological replicates each analysed in two technical replicates.

The Shapiro–Wilk test was used to assess the normality (*P* > 0.05) of data distribution. The Bartlett test was applied to test the homogeneity (*P* > 0.05) of variances. For data that met both criteria, analysis of variance was performed by applying the ANOVA test (*P* < 0.05) followed by Tukey post‐hoc test for multiple pairwise comparisons between group means. When homogeneity and normality assumptions were violated, the Kruskal–Wallis test (*P* < 0.05) followed by Dunn test (*P* < 0.05) were performed for multiple comparisons. Statistical analyses were performed using R software (R Core Team 2023). Principal Components Analysis (PCA) was conducted using *prcomp* function, and the first two principal components were used to interpret sample distribution and clustering. Scores and loading plots were generated with the *factoextra* package. Pearson correlation coefficients were computed between all phytochemical data from the 10 Brazilian samples and meteorological or environmental parameters (Tables S1 and S2). The correlation matrixes (Tables S4 and S5) were obtained using the *rcorr()* function from the *Hmisc* package. In addition to correlation coefficients, *P*‐values were calculated to determine statistical significance. The formatted correlation matrix was displayed using the *kable()* function from the *knitr* package. Heatmaps (Fig. 6a,b) were generated to graphically visualize correlation matrices using the *ggplot2* package.

## RESULTS

### Phenolic acid and alkaloid content

**Fig. 1:**
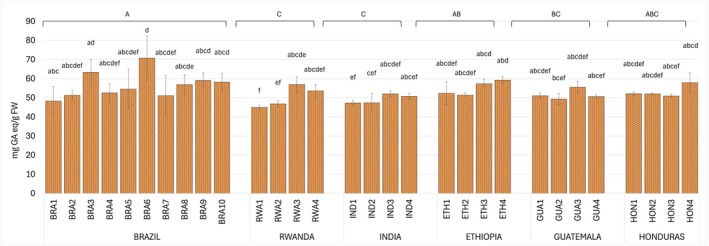
Total polyphenol content in green coffee bean samples from Brazil (BRA1‐10), Rwanda (RWA1‐4), India (IND1‐4), Ethiopia (ETH1‐4), Guatemala (GUA1‐4), and Honduras (HON1‐4). Data are mean (*n* = 3) ± SD. Uppercase letters on histograms indicate statistically different groups for countries and lowercase for samples, determined by Kruskal–Wallis test followed by Dunn's test (*P* < 0.05).

The Folin‐Ciocalteau assay was used to determine the total polyphenol content of the extracts (Fig. 1) that ranged from 44.8 mg GAeq g^−1^ FW (RWA1) to 70.7 mg GAeq g^−1^ FW (BRA6) indicating high variability in polyphenol content across the samples. Brazilian coffee beans had the highest polyphenol content among all countries, with an average of 56.6 mg GAeq g^−1^ FW. Specifically, Brazilian samples contained higher polyphenol levels compared to the average of other origins, with 11% more than Rwandan samples (50.1 mg GAeq g^−1^ FW), 13% more than Indian (49.3 mg GAeq g^−1^ FW), 3% more than Ethiopian (55.1 mg GAeq g^−1^FW), 9% more than Guatemalan (51.6 mg GAeq g^−1^ FW), and 6% more than Honduran samples (53.2 mg GAeq g^−1^ FW) (Fig. 1).

**Fig. 2:**
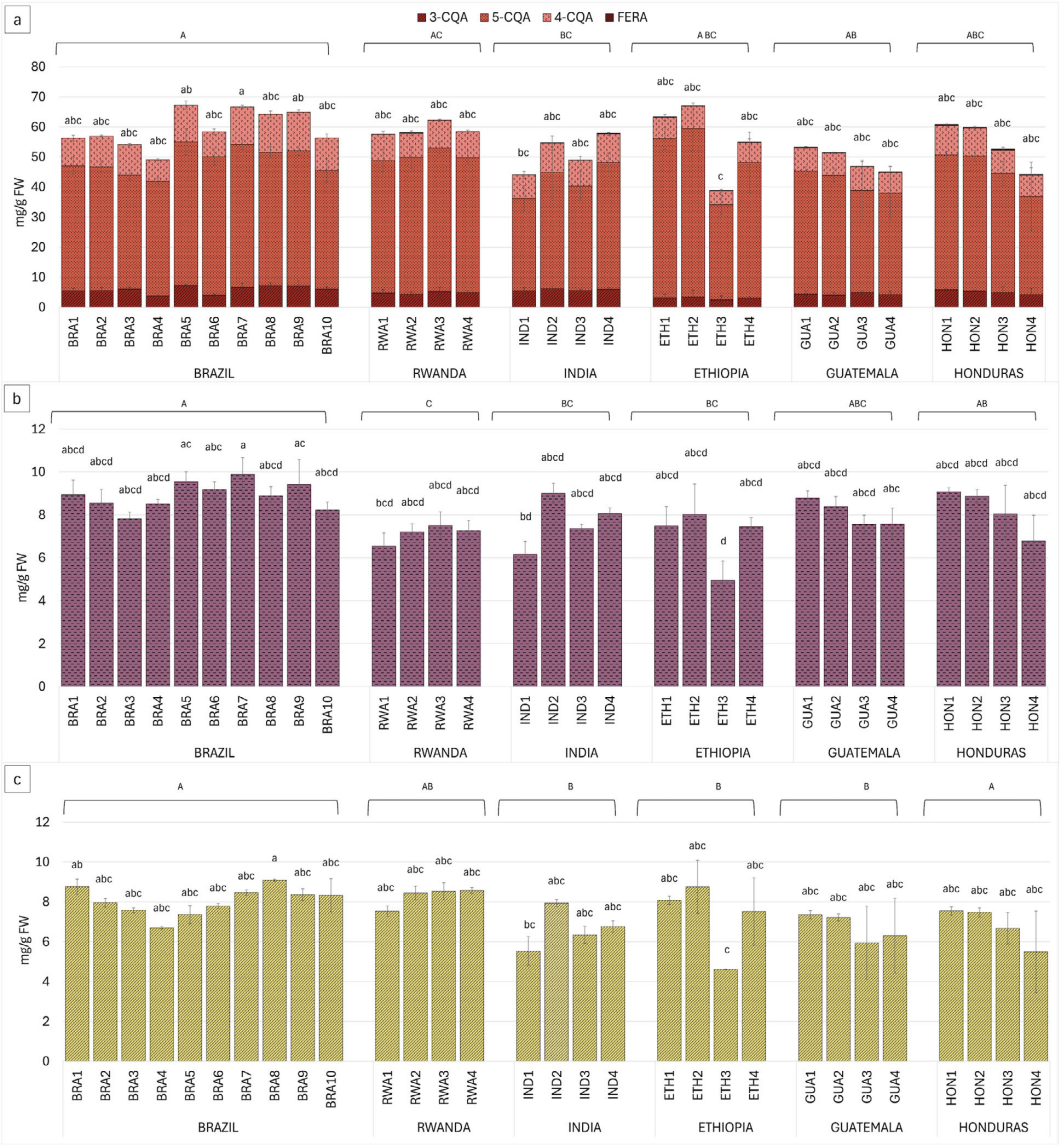
(a) HPLC‐DAD quantification of 3‐O‐caffeoylquinic acid (3‐CQA), 5‐O‐caffeoylquinic acid (5‐CQA), 4‐O‐caffeoylquinic acid (4‐CQA) and ferulic acid (FERA) (b), caffeine and (c) trigonelline contens in green coffee bean samples from Brazil (BRA1‐10), Rwanda (RWA1‐4), India (IND1‐4), Ethiopia (ETH1‐4), Guatemala (GUA1‐4), and Honduras (HON1‐4). Data are mean (*n* = 3) ± SD. Uppercase and lowercase letters on histograms indicate statistically different groups for countries, and lowercase for samples determined by Kruskal–Wallis test followed by Dunn's test (*P* < 0.05).

All chlorogenic acid isomers were detected by HPLC‐DAD in all samples, with 5‐O‐caffeoylquinic acid (5‐CQA, chlorogenic acid) having the highest concentration (Fig. 2a), on average eight‐fold higher than the 3‐O‐caffeoylquinic acid (3‐CQA, neochlorogenic acid) isomer and five‐fold higher than the 4‐O‐caffeoylquinic acid (4‐CQA, cryptochlorogenic acid) isomer. Ethiopian samples exhibited slight differences in isomer ratios (1:7:2 for 3‐CQA:5‐CQA:4‐CQA) compared to other countries, which showed ratios of 1:4:2 for India and Brazil and of 1:5:2 for Rwanda, Guatemala, and Honduras, respectively. Samples from Brazil, Rwanda, India, Guatemala, and Honduras had relatively homogeneous CQA levels, with averages ranging from 49.6 mg g^−1^ FW of Guatemala to 57.7 mg g^−1^ FW of Brazil. Ferulic acid (FERA) was absent in Brazilian samples but detected in all other countries (Fig. 2a). The lowest average concentration was found in Indian samples (0.19 mg g^−1^ FW), while the highest was in Honduran samples (0.33 mg g^−1^ FW).

**Fig. 3:**
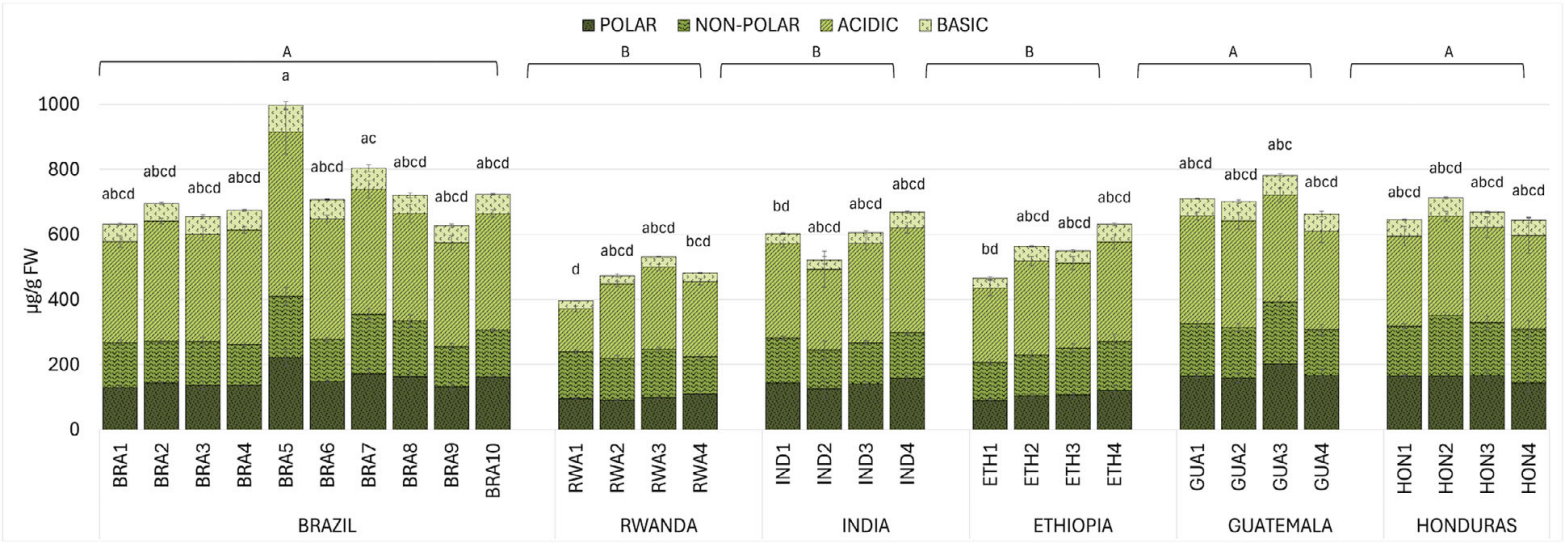
Free amino acid content in green coffee bean samples from Brazil (BRA1‐10), Rwanda (RWA1‐4), India (IND1‐4), Ethiopia (ETH1‐4), Guatemala (GUA1‐4), and Honduras (HON1‐4). POLAR (ser, thr, tyr), NON‐POLAR (gly, ala, pro, val, met, ile, leu, phe), ACIDIC (asp, glu), BASIC (hys, arg, lys). Data are mean (*n* = 3) ± SD. Uppercase and lowercase letters on histograms indicate statistically different groups for countries (ANOVA followed by Tukey's post‐hoc test, *P* < 0.05) and samples (Kruskal–Wallis test followed by Dunn's test, *P* < 0.05), respectively. See Table S3 for detailed sample amino acid data.

Caffeine (CAF) (Fig. 2b) exhibited varying concentrations across the groups, with Brazilian coffee having the highest average concentration (8.9 mg g^−1^ FW). Rwanda (average 7.1 mg g^−1^ FW), Guatemala (8.0 mg g^−1^ FW), and Honduras (8.1 mg g^−1^ FW) beans showed relatively consistent levels within each country. In contrast, greater CAF variability was detected within samples of India and Ethiopia (average content of 7.5 mg g^−1^ FW and 6.9 mg g^−1^ FW, respectively). Rwandan coffee had the highest trigonelline (TRI) average concentration (8.3 mg g^−1^ FW) followed by Brazil (8.0 mg g^−1^ FW) (Fig. 3b). India, Ethiopia, Guatemala and Honduras showed no significantly different TRI levels among countries with average levels ranging from 6.5 mg g^−1^ FW for India to 7.1 mg g^−1^ FW for Guatemala.

### Sucrose

The highest sucrose (SUC) content was detected in Ethiopian samples (average 10.9% (w/w) corresponding to 109 mg g^−1^ FW) (Table 1), followed by Brazilian samples (9.6% w/w). No significant differences were measured among SUC levels of Rwanda (9.2% w/w), Guatemala (9.4% w/w), and Honduras (8.6% w/w) samples, while Indian samples had the lowest average SUC content (8.3% w/w).

**Table 1:** Sucrose concentration (% w/w) in green coffee bean samples from Brazil (BRA1‐10), Rwanda (RWA1‐4), India (IND1‐4), Ethiopia (ETH1‐4), Guatemala (GUA1‐4), and Honduras (HON1‐4).

country	sample	sucrose (% w/w)	average (% w/w)
Brazil	BRA1	9.6 ± 0.5^ab^	9.6 ± 1.0^A^
	BRA2	9.9 ± 0.1^ab^	
	BRA3	10.7 ± 0.1^ab^	
	BRA4	7.6 ± 0.0^ab^	
	BRA5	9.1 ± 0.0^ab^	
	BRA6	9.6 ± 0.2^ab^	
	BRA7	10.4 ± 0.1^ab^	
	BRA8	8.2 ± 0.0^ab^	
	BRA9	9.6 ± 0.3^ab^	
	BRA10	10.6 ± 0.3^ab^	
Rwanda	RWA1	8.3 ± 1.0^ab^	9.2 ± 1.0^AC^
	RWA2	8.9 ± 1.1^ab^	
	RWA3	9.1 ± 0.6^ab^	
	RWA4	10.7 ± 0.7^ab^	
India	IND1	7.3 ± 0.8^b^	8.3 ± 1.4^C^
	IND2	8.2 ± 0.5^ab^	
	IND3	10.1 ± 0.3^ab^	
	IND4	7.4 ± 0.7^b^	
Ethiopia	ETH1	10.5 ± 0.4^ab^	10.9 ± 0.3^B^
	ETH2	10.9 ± 0.3^ab^	
	ETH3	10.9 ± 0.1^a^	
	ETH4	11.2 ± 0.6^a^	
Guatemala	GUA1	10.1 ± 0.2^ab^	9.54 ± 0.8^AC^
	GUA2	8.7 ± 1.2^ab^	
	GUA3	9.0 ± 0.1^ab^	
	GUA4	9.5 ± 0.2^ab^	
Honduras	HON1	8.5 ± 0.2^ab^	8.6 ± 0.4^AC^
	HON2	8.5 ± 0.1^ab^	
	HON3	8.5 ± 0.1^ab^	
	HON4	9.1 ± 0.4^ab^	

Data represent mean (*n* = 3) ± SD. Uppercase and lowercase letters indicate statistically different groups, respectively, for countries and samples (Kruskal–Wallis test followed by Dunn's test, *P* < 0.05).

### Free amino acid content

All targeted free amino acids were detected and quantified by HPLC‐FLD in all samples (Fig. 3, Table S3), except for cystine dimers (cysteine equivalent) (Fig. 3).

For each sample, amino acid contents were grouped as follows: POLAR (ser, thr, tyr), NON‐POLAR (gly, ala, pro, val, met, ile, leu, phe), ACIDIC (asp, glu), BASIC (hys, arg, lys). Amino acids were grouped based on the chemical properties of their side chains (R‐groups), which influence their solubility and interactions in biological systems.

The highest POLAR average amino acid content was detected in Brazilian beans (154.2 μg g^−1^ FW) while lowest levels were measured in Rwandan and Ethiopian samples (98.6 μg g^−1^ FW and 105.0 μg g^−1^ FW, respectively). Samples from Guatemala and Honduras had high concentrations of NON‐POLAR amino acids, with averages corresponding to 161.9 μg g^−1^ FW and 166.8 μg g^−1^ FW, respectively. High concentrations of ACIDIC amino acids, 48% of the total amino acid content, were consistently observed across all samples. Brazilian samples had, on average, significantly higher BASIC amino acid values (59.5 μg g^−1^ FW) compared to other countries, with lowest concentration detected in Rwanda (27.3 μg g^−1^ FW). The Brazilian sample BRA5 exhibited the highest concentration in each amino acid group, compared to all other samples, with a total content of 996.9 μg g^−1^ FW, significantly exceeding the average concentrations of the other Brazilian samples (692.9 μg g^−1^ FW).

### Biogenic amine content

**Fig. 4:**
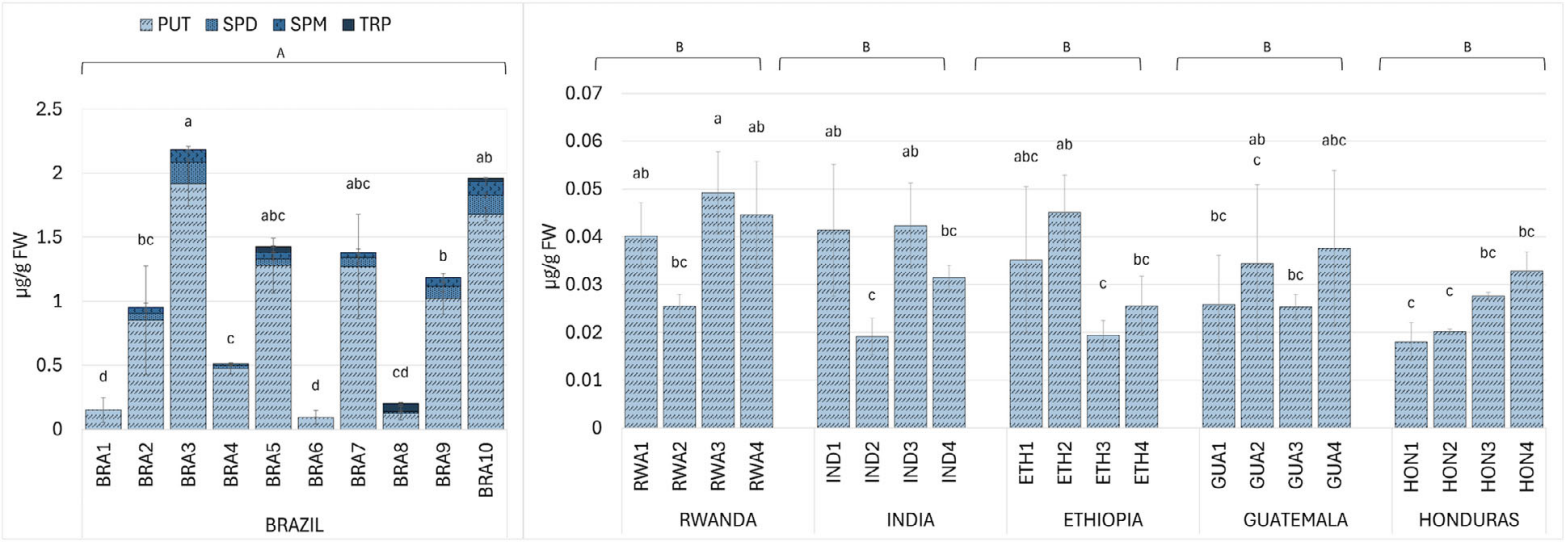
Free biogenic amines in green coffee bean samples from Brazil (BRA1‐10), Rwanda (RWA1‐4), India (IND1‐4), Ethiopia (ETH1‐4), Guatemala (GUA1‐4), and Honduras (HON1‐4). Putrescine (PUT), Spermidine (SPD), Spermine (SPM), and Tryptamine (TRP). Data are mean (*n* = 3) ± SD. Uppercase and lowercase letters on histograms indicate statistically different groups for countries (ANOVA test followed by Tukey post‐hoc test, *P* < 0.05) and samples (Kruskal–Wallis test followed by Dunn test, *P* < 0.05), respectively.

The HPLC‐FLD analysis of coffee green beans revealed the presence of three biogenic amines at higher concentrations: putrescine (PUT), spermidine (SPD), and spermine (SPM) (Fig. 4). Tryptamine (TRP) was detected in only three Brazilian samples, with a concentration of 0.04 μg g^−1^ FW in BRA8. The other investigated amines were not detected. PUT was the most abundant amine in all samples, accounting for almost 90% of the total identified amine content. Its concentration showed the most significant variability in Brazilian samples, with a minimum of 0.09 μg g^−1^ FW in sample BRA6 and a maximum of 1.92 μg g^−1^ FW in sample BRA3. SPD and SPM were detected in almost all Brazilian samples, with maximum levels, respectively, of 1.83 μg g^−1^ FW in sample BRA3 and of 0.11 μg g^−1^ FW in sample BRA10. In samples from the other five countries, PUT was the only detected polyamine, but its concentration was significantly lower than in the Brazilian samples (<4%). The average concentrations ranged from 0.04 μg g^−1^ FW in Rwanda to 0.02 μg g^−1^ FW in Honduras beans, with no significant differences among different countries (Fig. 4). SPD and SPM were present in trace amounts, below the technical limit of quantification.

### PCA score plot and loading plot

**Fig. 5:**
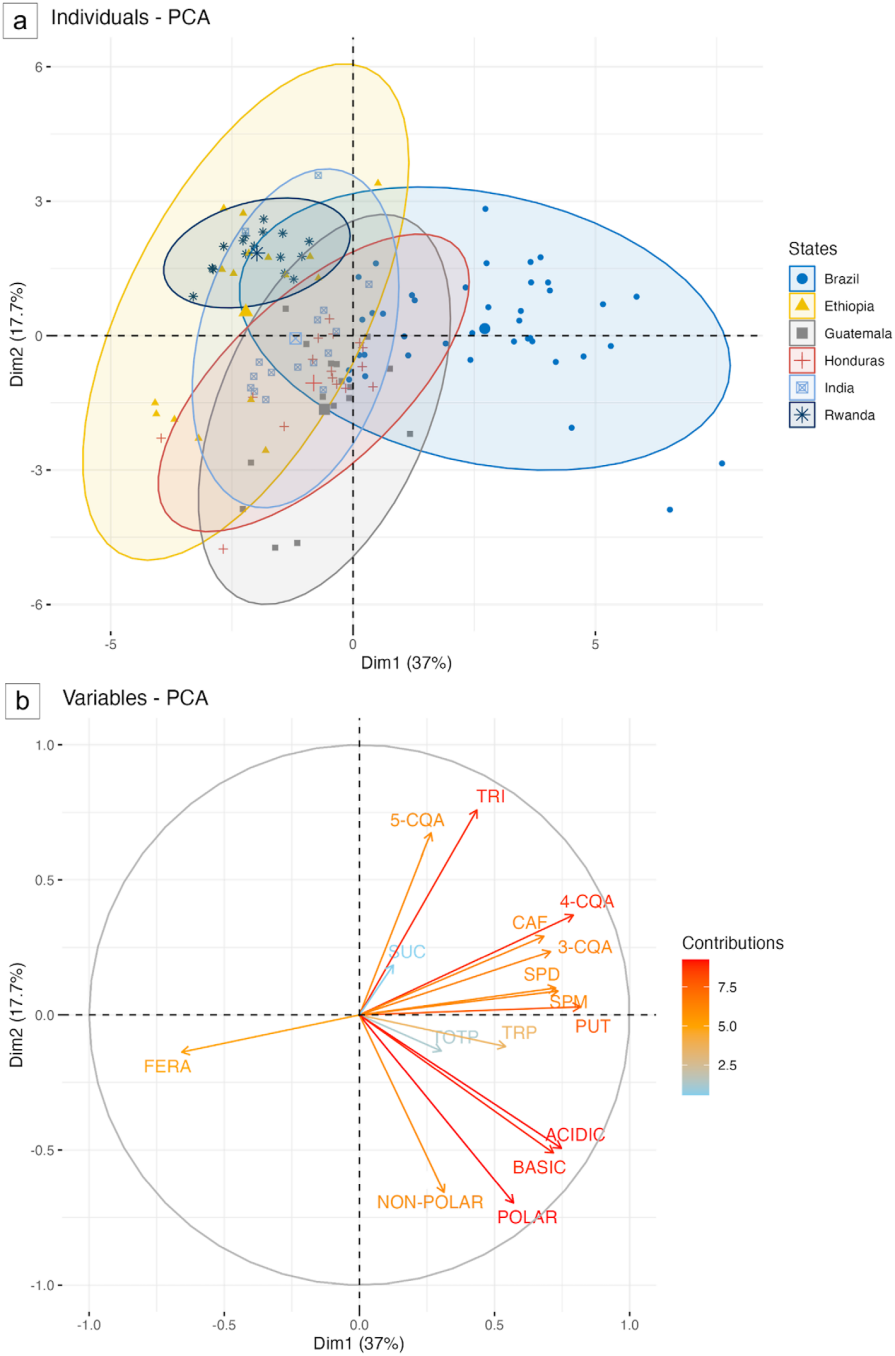
(a) PCA score plot: each point represents sample replicates and ellipses indicate 95% confidence intervals around the mean for each state. (b) PCA loadings plot illustrating contributions of each variable to the first two principal components. The coloured gradient represents the magnitude of each variable's contribution. The analyses were conducted using R (R Core Team 2023). The *prcomp()* function was used for principal component computation. The *factoextra* package was employed for visualization in both score plot and loadings plot. 3‐CQA, 5‐CQA, 4‐CQA: 3‐, 5‐, 4‐O‐caffeoylquinic acid; CAF, caffeine; POLAR, NON‐POLAR, ACID, BASIC, free polar, non‐polar, acidic and basic amino acids; PUT, putrescine; SPD, spermidine; SPM, spermine; SUC, sucrose; TOTP, total polyphenols; TRI, trigonelline; TRP, tryptamine.

A PCA was employed to visualize patterns and assess variability within the collected data, allowing the identification of key principal components and their contribution to the overall variance (Fig. 5). The distribution of individual samples is displayed with ellipses centered around the origin for each country. The ellipses represent the 95% confidence interval, providing a visual indication of the grouping of samples by countries based on their proximity to the first two principal components. PC1 and PC2, together accounting for the majority of the data's variance, were selected for further analysis as they provide a clear overview of the relationships between the variables.

The PCA score plot based on all phytochemical data (Fig. 5a) shows a distinct cluster for Brazilian samples with some overlap, partially discriminating Brazilian samples from other countries. Brazil and Ethiopia samples are widely spread, indicating significant variability within countries. The presence of outliers, which appear outside the confidence ellipses, may indicate sample natural variability. Rwandan group forms a compact cluster, indicating greater homogeneity while significantly overlapping with Ethiopian samples. Honduras and Guatemala ellipses mostly overlap and exhibit similar variability in their chemical characteristics indicating comparable patterns of variation with a subtle difference in the overall concentration of the samples.

To further investigate the contribution of specific compounds, three additional targeted PCAs were performed on specific subsets of metabolites (Fig. S1). The first targeted PCA, focusing on 3‐CQA, 5‐CQA, and 4‐CQA (Fig. S1a), revealed a strong clustering pattern, distinctly separating Ethiopian samples from those of other countries. The second targeted PCA, analysing caffeine and trigonelline levels (Fig. S1b), similarly differentiated Ethiopian samples while also forming a distinct cluster for Rwandan samples, which partially overlap with those from Brazil, India, and Ethiopia. In contrast, samples from Honduras and Guatemala exhibited a complete overlap. A third PCA was conducted on data of the 16 individual free amino acids (Fig. S1c) to evaluate their specific contributions to sample differentiation. This analysis revealed distinct clustering of Brazilian samples, whereas Rwandan, Indian, and Ethiopian samples exhibited substantial overlap. Honduran samples partially overlapped with those from Guatemala, Ethiopia, and India.

The PCA loadings plot (Fig. 5b) illustrates the contributions of the different variables to PC1 and PC2. Among them, 4‐CQA, TRI, PUT, POLAR, ACIDIC and BASIC are the most significant contributors to the overall dataset variance. Specifically, PUT, SPD, SPM, 4‐CQA, ACIDIC and BASIC have the strongest loadings on PC1, all positively associated with this principal component. TRI and 5‐CQA primarily contribute to PC2 variation, showing a positive association with it. In contrast, POLAR, NON‐POLAR as well as BASIC and ACIDIC variables exhibit the most significant negative contributions to PC2. Notably, FERA showed a distinct contribution compared to other metabolites. Variables clustered closely together indicate a positive correlation, whereas vectors forming a nearly 180° angle, like 3‐CQA and FERA, suggest a negative correlation between these variables. The length of the arrows in the plot visually represents the strength of each variable contribution, with longer arrows indicating stronger effects.

### Correlation analysis

**Fig. 6:**
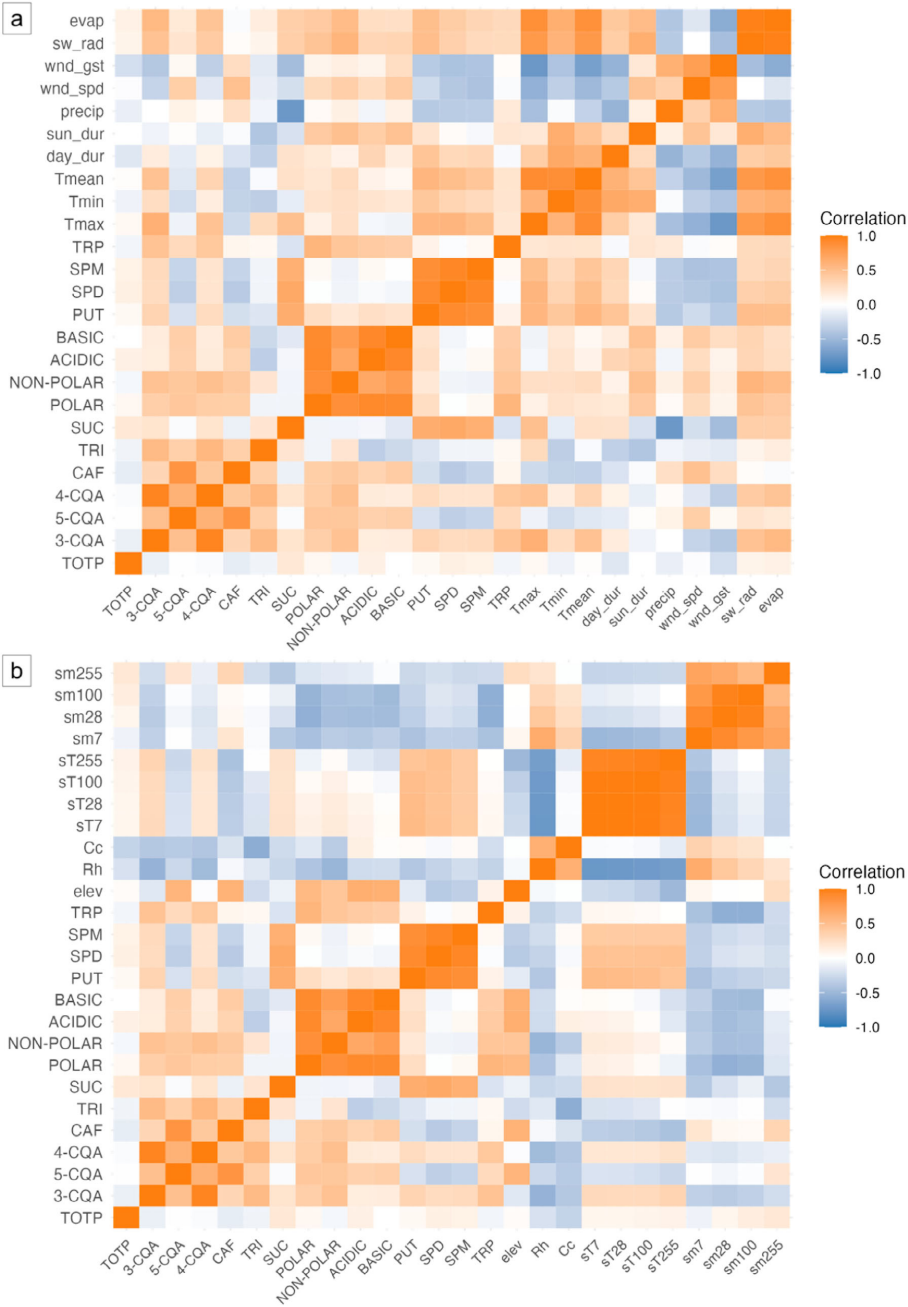
Heatmaps showing Pearson coefficients indicating positive (orange) to negative (light blue) correlations between phytochemical data of green coffee bean samples from Brazil (BRA1‐10) and cultivation sites (a) meteorological parameters (Table S1) or (b) environmental parameters (Table S2). Phytochemical compounds: TOTP, total polyphenols; 3‐CQA, 5‐CQA, 4‐CQA: 3‐, 5‐, 4‐O‐caffeoylquinic acid; CAF, caffeine; TRI, trigonelline; SUC, sucrose; POLAR, NON‐POLAR, ACID, BASIC, free polar, non‐polar, acidic and basic amino acids; PUT, putrescine; SPD, spermidine; SPM, spermine; TRP, tryptamine. Meteorological parameters: Tmax, Tmin, Tmean: maximum, minimum and mean temperatures; day_dur, daylight duration; sun_dur, sunshine duration; precip, rainfall; wnd_spd, average wind speed; wnd_gst, maximum wind gusts; sw_rad, solar shortwave radiation; evap, evapotranspiration. Environmental parameters: elev, elevation; Rh, relative humidity; Cc, cloud cover; sT7, sT28, sT100, sT255: soil temperature at 7, 28, 100, 255 cm depth; sm7, sm28, sm100, sm255: soil moisture at 7, 28, 100, 255 cm depth. See Tables S4 and S5 for detailed numerical Pearson correlation indices.

Meteorological and environmental data for the maturation period (April to June 2021) were obtained based on the exact cultivation locations, which were available only for Brazilian samples (Tables S1 and S2). Pearson correlation indices were calculated (R Core Team 2023) between all phytochemical data from the 10 Brazilian samples and meteorological or environmental parameters (Tables S1 and S2) to highlight how compound concentrations change in response to growing conditions. The analysis focused on general trends rather than relationships among individual samples. Heatmaps (Fig. 6a,b) present a graphical representation of the Pearson's correlation values, with a colour scale ranging from blue to orange, indicating negative to positive correlations, while white colour represents weak or no correlation. The detailed Pearson correlation indices are reported in Tables S4 and S5.

Among chlorogenic acid isomers, data indicated a highly significant positive correlation between 5‐CQA levels and elevation (*P* ≤ 0.001) (Fig. 6a, Table S4). 3‐CQA and 4‐CQA showed similar trends with positive correlations (*P* ≤ 0.01–0.001) with shortwave radiation (sw_rad), maximum and mean temperatures (Tmax, Tmean), and evapotranspiration (evap), while a negative correlation with relative humidity (Rh), cloud cover (Cc), and wind gusts (wnd_gst) (Fig. 6a,b; Tables S4 and S5). Total polyphenol levels (TOTP) showed no significant correlations with the analysed parameters. CAF showed a strong positive correlation with elevation (elev, *P* ≤ 0.001) but was negatively associated with minimum and mean temperatures (Tmin, Tmean), as well as with soil temperatures at different depths (sT7–sT255; *P* ≤ 0.05–0.01); TRI was negatively correlated with Tmin, daylight duration and sunshine duration (Fig. 6a,b; Tables S4 and S5). SUC content showed significant positive correlation with Tmax (*P* ≤ 0.01) and negative correlation with precipitation (precip, *P* ≤ 0.001) and wind gusts (*P* ≤ 0.01).

Several meteorological and environmental parameters correlated with amino acid content. As examples, POLAR amino acids were positively correlated, among others, with elevation (*P* ≤ 0.001), and negatively correlated with soil moisture at different depths (sm7–sm100, *P* ≤ 0.01 to 0.001), while NON‐POLAR amino acids were positively correlated with shortwave radiation and evapotranspiration (*P* ≤ 0.001) and negatively correlated soil moisture (sm7–sm100; *P* ≤ 0.05 to 0.001) (Fig. 6a,b; Tables S4 and S5).

Highly significant correlations were found between PUT, SPD, SPM and meteorological variables, particularly temperature, shortwave radiation, evapotranspiration, rainfall, and wind parameters (Fig. 6a, Table S4). PUT showed positive correlations (*P* ≤ 0.001) with Tmax, Tmin, and Tmean, shortwave radiation, and evapotranspiration. In contrast, PUT was negatively correlated with rainfall and wind gusts (*P* ≤ 0.05). SPD was positively associated with Tmax and Tmean (*P* ≤ 0.001), shortwave radiation and evapotranspiration (*P* ≤ 0.05), and negatively correlated with rainfall and wind speed (*P* ≤ 0.05). A similar trend was detected for SPM (Fig. 6a, Table S4). Significant correlations were also observed between PUT, SPD, and SPM with environmental parameters, including elevation, relative humidity (Rh), cloud cover (Cc), soil temperature (sT), and soil moisture (sm). The three PAs showed a highly significant positive correlation with soil temperatures at different depths (sT7–sT255, *P* ≤ 0.01–0.001) while, on the other hand, a negative correlation with elevation (*P* ≤ 0.05), and/or soil moisture (sm7–sm255, *P* ≤ 0.05–0.01) (Fig. 6b, Table S5).

## DISCUSSION

The main aim of this research was to correlate the content of different phytochemicals in Arabica green coffee beans with their origin countries, which presumably have different environmental conditions for coffee plant growth. The classes of compounds examined were selected based on previous evidence suggesting that their biosynthetic levels are influenced by environmental factors, consequently affecting the sensory quality of coffee beans.

Data highlighted some differences in the phytochemical levels of green coffee bean samples from different countries; however, it was not possible to fully distinguish all sample groups based on their geographic origins. Nevertheless, certain compound levels appeared to be correlated with key meteorological and environmental factors that influence the biochemical composition of coffee beans.

The results for total polyphenols revealed significant variation among coffee bean samples from different geographic origins. Brazilian samples had the highest overall average total polyphenol levels (56.6 mg GAeq g^−1^ FW), while Indian samples had the lowest (49.3 mg GAeq g^−1^ FW) (Fig. 1). The average total polyphenol content measured across all samples from all countries (53.5 mg GAeq g^−1^ FW) is similar to that reported by Pérez‐Hernández *et al*. (2012) (55.84 mg GAeq g^−1^ FW) in green Arabica beans.

The detected average content of total chlorogenic acids (CQA) ranged from 49.6 mg g^−1^ FW for Guatemala to 62.2 mg g^−1^ FW for Ethiopia (Fig. 2a), which fall within the range previously reported in literature (Ky *et al*. 2001; Farah & Donangelo 2006). The differences in isomer ratio observed in Ethiopian samples, characterized by higher concentrations of 5‐CQA and lower concentrations of 3‐CQA and 4‐CQA compared to samples from other countries, could be attributed to the accumulation patterns of chlorogenic acid isomers. Specifically, 5‐CQA isomer predominantly accumulates in immature coffee beans and decreases in the final stages of maturation, while 3‐CQA and 4‐CQA progressively increase throughout maturation (Oestreich‐Janzen 2013; Cheng *et al*. 2016). This suggests that the Ethiopian beans in this study may have been harvested at different stages of ripeness, leading to variations in maturation levels. The low concentrations of ferulic acid detected (Fig. 2a), can be attributed its binding to cell wall components, with only small amounts generally present in the free form (Farah & Donangelo 2006).

The caffeine (CAF) concentration data follow a similar trend to that reported for chlorogenic acids (Fig. 2b). Ethiopian samples had the lowest average CAF concentration (6.9 mg g^−1^ FW) among all countries of origin, consistent with findings by Wale *et al*. (2024) on Arabica green coffee beans grown in different Ethiopian regions. The overall CAF content in this study (Fig. 2b), was 15% to 50% lower than the typical range for Arabica coffee (Oestreich‐Janzen 2013). These variations in concentration could potentially reflect environmental growing conditions (Wale *et al*. 2024), such as altitude, which has been linked to CAF content in coffee bean, with air temperature controlling biosynthesis and degradation ratio in coffee beans during seed development (Joët, Salmona, *et al*. 2010). However, given that the detailed location or climate data were not available for this country, a potential influence of seed maturity or other post‐harvesting processing parameters could not be ruled out. Nevertheless, as previously mentioned, CAF content is primarily determined by genetic factors (Oestreich‐Janzen 2013; Cheng *et al*. 2016), thus the observed variation likely reflects a combination of the stated factors. The trigonelline (TRI) concentration across coffee samples from different geographic origins (Fig. 2c) averaged 7.4 mg g^−1^ FW, aligning with previously reported values (Ky *et al*. 2001; Oestreich‐Janzen 2013; Wale *et al*. 2024). Rwandan and Brazilian coffee exhibited the highest average TRI content, while Ethiopian samples showed the greatest variability (Fig. 2c). These findings are consistent with previous studies reporting significant TRI variability in coffee samples from major coffee‐producing regions in southwest Ethiopia (Sualeh *et al*. 2020). TRI biosynthesis follows a pattern similar to CAF, increasing in young seeds and declining in mature tissues. Like CAF, the genetic diversity plays a crucial role in TRI level variations (Wale *et al*. 2024).

Sucrose (SUC) content across all samples (Table 1) ranged between 7.3% and 11.2% (w/w), aligning with previously reported SUC values in Arabica coffee beans (6%–11% w/w) (Ky *et al*. 2001; Campa *et al*. 2004; Stredansky *et al*. 2018).

Data on all free amino acid contents (Fig. 3, Table S3) showed lower concentrations in all analysed samples compared to previous studies on Arabica green coffee beans (Arnold *et al*. 1994; Casal *et al*. 2003; Lonzarich *et al*. 2012). However, despite these differences in overall concentration, the relative proportions of individual amino acids remained generally consistent with previous reports (Arnold *et al*. 1994; Lonzarich *et al*. 2012). Earlier studies on Arabica coffee beans identified glutamic acid as particularly abundant, accounting for approximately 40% of the total free amino acid content (Lonzarich *et al*. 2012). This is in agreement with the present findings, where glutamic acid was the predominant amino acid in all samples, followed by aspartic acid, together representing on average of about 48% of the total free amino acids (Fig. 3, Table S3). While Arnold *et al*. (1994) suggested that glutamic acid level can fluctuate significantly during the postharvest storage phase, its concentration can also vary considerably depending on the geographic origin of the coffee beans. Increasing evidence highlights the role of amino acids and their derivatives in regulating pathways involved in plant responses to abiotic stress (Heinemann & Hildebrandt 2021).

Among the studied free biogenic amines (BAs), putrescine (PUT) was the most abundant in all samples; spermidine (SPD) and spermine (SPM) were detected at measurable levels only in Brazilian samples (Fig. 4). Tryptamine (TRP) was detected only in three Brazilian samples at low concentrations. Its presence, only reported in a few studies on unroasted Arabica beans, has been associated with immature beans typically considered lower quality due to their negative impact on sensory attributes (Oliveira *et al*. 2005; Sridevi *et al*. 2009).

The total BA levels measured across Brazilian samples (from 0.09 to 1.92 μg g^−1^ FW; Fig. 4), were significantly higher than those in samples from the other five countries, whose concentrations were less than 4% of the highest Brazilian sample. Furthermore, these values were lower than those reported in a previous study on Arabica coffee samples from Brazil (Cirilo *et al*. 2003), where PUT average concentrations were 10 times higher (9 μg g^−1^) than those here reported in Brazilian samples (0.89 μg g^−1^ FW; Fig. 4). Among BA, polyamines (PAs) are known to be involved in cell division and growth but also to play a role in plant adaptation to environmental growth conditions (Chen *et al*. 2019; Shao *et al*. 2022). The differences in PA concentrations across samples from different countries (Fig. 4), may therefore reflect the combined influences of varying climatic and soil growth parameters, as well as to specific environmental stresses such as drought, heat, chilling, and salt stress, together with genetic variation among coffee varieties (Bagni *et al*. 2006; González‐Hernández *et al*. 2022; Shao *et al*. 2022; Blázquez 2024). These variations can be interpreted as part of broader metabolic adjustments to environmental conditions, potentially linked to adaptive processes in coffee.

The PCA revealed distinct clustering patterns among the samples, highlighting both similarities and variability across samples from different countries only partially related to the geographic provenience (Fig. 5a). The partial separation of Brazilian samples suggests that the selected bioactive compounds contribute to regional differentiation. Overlapping clusters indicate shared biochemical characteristics between the countries. The broad dispersion of Brazilian and Ethiopian samples reflects substantial within‐country variability, likely influenced by environmental or genetic factors. In contrast, the compact grouping of Rwandan and Indian samples suggests greater homogeneity, while their overlap with Ethiopian samples points to similarities in biochemical composition. The close alignment of Honduran and Guatemalan samples further supports a comparable chemical profile, with only slight variations in concentration levels. The targeted PCA results on specific subsets of compounds suggest that specific metabolite classes drive regional variation in coffee samples. Recently, Tieghi *et al*. (2024) reported that phenolic and alkaloid markers such as chlorogenic acids, caffeine, and trigonelline effectively differentiate Brazilian coffee samples among different regions indicating distinct chemical profiles influenced by both geographic origin and processing methods. In the present study, chlorogenic acids, caffeine and trigonelline (Figs. S1a, b) appear to be key markers for Ethiopian coffee, as these samples consistently formed a separate cluster. This may suggest region‐specific biosynthesis or post‐harvest processing differences affecting these compounds. Free amino acid profiles (Fig. S1c) showed a contribution to the differentiation of Brazilian samples, but a more extensive overlap across other regions, suggesting that these compounds may be less influential in geographic differentiation or subject to greater environmental variability. Overall, these findings highlight the potential of specific metabolites as biomarkers for geographic differentiation. The PCA loadings plot highlights the key compounds driving these patterns and the varying arrow lengths show the different impact of each variable in defining the observed clustering (Fig. 5b). The strong influence of PUT, SPD, SPM, 4‐CQA, ACIDIC, and BASIC on PC1 suggests their critical role in shaping overall variance, possibly indicating these compounds as the most susceptible to the different environmental growth conditions. On the other hand, TRI, 5‐CQA, POLAR, NON‐POLAR, ACIDIC and BASIC free amino acids mainly influenced PC2, with roles in distinguishing sample groups (Fig. 5b).

For Brazilian samples, Pearson correlation indices could be calculated between all phytochemical data from the 10 Brazilian samples and the collected meteorological or environmental parameters (Fig. 6a,b; Tables S4 and S5). However, since detailed location information was not available for non‐Brazilian samples, the environmental interpretation for these samples was not possible. Including precise location data from all countries in this work would have provided a more complete understanding of how environmental factors influence the phytochemistry of Arabica coffee beans. The correlation analyses revealed that higher temperatures and increased radiation levels emerged as key drivers, showing strong positive associations with the PUT, SPD, and SPM levels (Fig. 6a, Table S4), suggesting that these conditions may be linked to the accumulation of these bioactive compounds. Although all samples underwent the same post‐harvesting processes and were collected shortly afterward, these processing methods could also have partially influenced the levels of PAs in the beans. PAs are known to play a crucial role in plant tolerance to abiotic stress, including heat, by regulating their biosynthetic pathways at the transcriptional level (Shao *et al*. 2022; Blázquez 2024). Research on high‐temperature stress suggested that PAs contribute to heat tolerance by enhancing photosynthesis, increasing antioxidant capacity, and improving osmotic adjustment (Chen *et al*. 2019). Similarly, soil temperature was positively correlated with PUT, SPD, and SPM concentrations (Fig. 6b, Table S5) (Chen *et al*. 2019). Conversely, the negative correlations with rainfall and soil moisture align with studies showing that PA plays a crucial role in plant drought resistance by regulating stomatal function and osmotic balance. Increased PA synthesis has in fact been linked to enhanced plant drought stress tolerance (Chen *et al*. 2019; Blázquez 2024). Although there is no direct evidence, increased PA accumulation in stressed plants could promote greater PA transport or accumulation in seeds. Other than having crucial roles in plant stress responses, PAs are also essential regulators of reproductive development and embryogenesis (Chen *et al*. 2019), suggesting a potential role in seed development under stress conditions. Further studies are needed to clarify PA transport dynamics and metabolic regulation in coffee seeds under abiotic stress.

The strong positive correlation between CAF and 5‐CQA content and elevation (Fig. 6b, Table S5) suggests that altitude of coffee cultivation site influences their accumulation, possibly related to variations in temperature and potentially oxygen availability at higher elevations (Girma *et al*. 2020). However, previous studies on green coffee beans reported altitude as negatively correlated to CAF and 5‐CQA content (Tolessa *et al*. 2017; Girma *et al*. 2020). All the analysed Brazilian samples were grown at elevations ranging from 841 to 1088 m a.s.l. (Table S1), which are considerably lower than those studied by Tolessa *et al*. (2017) or comparable to the lowland group examined by Girma *et al*. (2020). In addition, CAF and 5‐CQA concentrations are also impacted by shade and harvest period since their biosynthesis and accumulation happen during early bean development (Oestreich‐Janzen 2013; Tolessa *et al*. 2017). Consequently, while altitude‐driven temperature variations play a crucial role, genetic factors, along with additional environmental and developmental conditions, further shape CAF and 5‐CQA accumulation patterns in coffee beans.

The detected positive correlation between SUC concentrations and maximum temperature (Tmax), as well as the negative correlation with precipitation levels (Fig. 6a,b, Tables S4 and S5), is in agreement with the previous literature, which reported higher SUC content in beans from non‐irrigated coffee plants grown in warmer climates or under drought stress conditions (Silva *et al*. 2005; Vinecky *et al*. 2017).

Amino acid accumulation is also strongly influenced by environmental factors (Trovato *et al*. 2021). The reported highly significant negative correlations between POLAR and NON‐POLAR amino acid groups and relative humidity or soil moisture (Fig. 6b, Table S5), emphasize the impact of water stress on their accumulation. Previous studies have shown that proline, a non‐polar amino acid, acts as a key osmoprotectant by increasing under osmotic stress to help maintain cellular stability (Suprasanna *et al*. 2014). Similarly, other non‐polar branched‐chain amino acids (Val, Leu, Ile) play a crucial role in dehydration tolerance by serving as alternative energy sources. When photosynthesis is limited, these amino acids are released through protein breakdown and directed into the TCA cycle to sustain respiration and energy production under stress conditions (Batista‐Silva *et al*. 2019). Additionally, phenylalanine (Phe) (non‐polar) and other aromatic amino acids play a role as precursors in the biosynthesis of secondary metabolites. Phe is converted into flavonoids, lignin, and other compounds that partially support plant defence and adaptation under environmental stress (Batista‐Silva *et al*. 2019). The investigation on stress‐associated metabolites in green coffee beans may provide indications on this plant's physiological responses during fruit development under varying environmental conditions. To complement this information, root and leaf tissues are being analysed to broaden the understanding of coffee plant metabolic responses to stress conditions and climate change.

## CONCLUSIONS

Overall, the above findings highlight the complex interplay between geographic provenance and environmental growth parameters in modulating bioactive compound accumulation in coffee green beans. The variations observed in phytochemical compounds, including total polyphenols, chlorogenic acids, caffeine, trigonelline, free amino acids, and biogenic amines, reflect the complex relationship among climatic, atmospheric, and soil parameters and plant metabolic responses. The PCA revealed distinct patterns among Arabica coffee samples, indicating partial separation of Brazilian samples from the other countries, and within‐country variability, in samples from both Brazil and Ethiopia. Key compounds such as putrescine, trigonelline, 4‐O‐caffeoylquinic acid and polar, acidic and basic amino acid groups, primarily drive the chemical variability among samples. The strong association between polyamine levels and temperature or moisture conditions highlights their role in plant stress responses. Additionally, the accumulation of specific polar and non‐polar free amino acids suggests potential mechanisms for osmotic and drought protection. These findings provide valuable insights into the biochemical composition of coffee beans and their adaptive responses to environmental factors. A deeper understanding of these variations can enhance the assessment of desirable sensory characteristics and improve market value, while contributing to optimize cultivation strategies in the face of climate change. Future studies should further investigate the genetic mechanisms underlying these metabolic shifts to enhance the adaptability of multiple coffee species and varieties.

## AUTHOR CONTRIBUTIONS

AT, MF, and LN conceptualized and designed the study. LN provided the samples for the experiments. IP, GB, SM, and EDA conducted the experiments and analysed the data. IP and GB wrote the initial draft, while AT, MF, and LN reviewed and edited the manuscript. AT supervised the project. All authors approved the submitted version.

## FUNDING INFORMATION

This study was carried out within the Agritech National Research Center and received funding from the European Union—NextGenerationEU (PIANO NAZIONALE DI RIPRESA E RESILIENZA (PNRR) – MISSIONE 4 COMPONENTE 2, INVESTIMENTO 1.4 – D.D. 1032 17/06/2022, CN00000022).

## CONFLICT OF INTEREST STATEMENT

The authors declare that there are no conflicts of interest related to this study.

## Supporting information


**Table S1.** Brazilian sample mean values for meteorological parameters related to the specific cultivation sites during the maturation period (April to June 2021).
**Table S2.** Brazilian sample mean values for environmental parameters related to the specific cultivation sites during the maturation period (April to June 2021).
**Table S3.** Free amino acid content (μg g^−1^ FW) in green coffee bean samples from Brazil (BRA1‐10), Rwanda (RWA1‐4), India (IND1‐4), Ethiopia (ETH1‐4), Guatemala (GUA1‐4), and Honduras (HON1‐4).
**Table S4.** Matrix including Pearson correlation coefficients between phytochemical data of green coffee beans from Brazil (BRA1‐10) and meteorological parameters of cultivation sites.
**Table S5.** Matrix including Pearson correlation coefficients between phytochemical data of green coffee beans from Brazil (BRA1‐10) and environmental parameters of cultivation sites.
**Fig. S1.** Principal Components Analysis (PCA) score plots for specific compound subsets: (a), 3‐, 5‐, 4‐caffeoylquinic acids; (b) caffeine and trigonelline; (c) free amino acids (ser, thr, tyr, gly, ala, pro, val, met, ile, leu, phe, asp, glu, hys, arg, lys).

## Data Availability

The original contributions presented in the study are included in the article/Supporting Information S1, further inquiries can be directed to the corresponding author.
