# Supplementary material for: Geographic provenance and environmental growing conditions as factors influencing phytochemical composition of Arabica green coffee beans
Source: Plant Biol (Stuttg). 2025 Nov 11;28(2):520–34. doi: 10.1111/plb.70136 (PMC12884032; doi:10.1111/plb.70136)
Supplement: Supplementary file 1 — Table S1. Brazilian sample mean values for meteorological parameters related to the specific cultivation sites during the maturation period (April to June 2021). Table S2. Brazilian sample mean values for environmental parameters related to the specific cultivation sites during the maturation period (April to June 2021). Table S3. Free amino acid content (μg g−1 FW) in green coffee bean samples from Brazil (BRA1‐10), Rwanda (RWA1‐4), India (IND1‐4), Ethiopia (ETH1‐4), Guatemala (GUA1‐4), and Honduras (HON1‐4). Table S4. Matrix including Pearson correlation coefficients between phytochemical data of green coffee beans from Brazil (BRA1‐10) and meteorological parameters of cultivation sites. Table S5. Matrix including Pearson correlation coefficients between phytochemical data of green coffee beans from Brazil (BRA1‐10) and environmental parameters of cultivation sites. Fig. S1. Principal Components Analysis (PCA) score plots for specific compound subsets: (a), 3‐, 5‐, 4‐caffeoylquinic acids; (b) caffeine and trigonelline; (c) free amino acids (ser, thr, tyr, gly, ala, pro, val, met, ile, leu, phe, asp, glu, hys, arg, lys). [file PLB-28-520-s001.pdf]

**Supplementary Material**

**Ilaria Pettazzoni[1], Giorgia Benati[1], Stefania Monari[1], Elisabetta De Angelis[2] Luciano Navarini[3], Maura Ferri[1*], Annalisa Tassoni[1].**

[1] Department of Biological, Geological, and Environmental Sciences, University of Bologna, Bologna, Italy

[2]Aromalab illycaffè S.p.A., Area Science Park, Padriciano 99, 34149 Trieste, Italy

[3]illycaffè S.p.A., Via Flavia 110, 34147 Trieste, Italy

*** Correspondence:**
Maura Ferri, maura.ferri@unibo.it

**Keywords:** Arabica coffee beans, Chlorogenic acids, Caffeine, Polyphenols, Biogenic amines**.**

**Table S1.** Brazilian samples mean values for meteorological parameters related to the specific cultivation sites during maturation period (April to June 2021). Maximum temperature (Tmax), minimum temperature (Tmin), mean temperature (Tmean), daylight duration (day_dur), sunshine duration (sun_dur), rainfall (precip), average wind speed (wnd_spd), maximum wind gusts (wnd_gst), shortwave radiation (sw_rad) and evapotranspiration (evap). Data were sourced from Visual Crossing Weather 2020-2021 database (Visual Crossing Corporation).

| Sample | Location | Tmax (°C) | Tmin (°C) | Tmean (°C) | day_dur (sec) | sun_dur (sec ) | precip (mm) | wnd_spd (km/h) | wnd_gst (km/h) | sw_rad (MJ/m²) | evap (mm) |
|---|---|---|---|---|---|---|---|---|---|---|---|
| BRA1 | Divisa Nova | 24.3 | 13.2 | 18.2 | 40217 | 35262 | 0.56 | 12.8 | 32.6 | 16.61 | 3.2 |
| BRA2 | Capelinha | 23.9 | 14.1 | 18.7 | 40861 | 35046 | 0.36 | 12.0 | 29.5 | 16.43 | 3.2 |
| BRA3 | Patos De Minas | 27.1 | 16.0 | 21.3 | 40708 | 36114 | 0.41 | 12.0 | 28.0 | 17.42 | 3.8 |
| BRA4 | Araponga | 22.0 | 14.5 | 17.8 | 40362 | 35995 | 0.93 | 13.5 | 34.8 | 16.18 | 2.9 |
| BRA5 | São Gotardo | 24.7 | 15.0 | 19.5 | 40590 | 36047 | 0.63 | 13.0 | 31.4 | 17.17 | 3.6 |
| BRA6 | Campestre | 22.7 | 12.5 | 17.2 | 40180 | 35497 | 0.54 | 13.7 | 31.7 | 16.56 | 3.2 |
| BRA7 | Tapira | 24.3 | 13.9 | 18.6 | 40483 | 36045 | 0.62 | 14.2 | 32.9 | 17.12 | 3.5 |
| BRA8 | Monte Santo De Minas | 25.0 | 14.6 | 19.3 | 40265 | 35649 | 0.82 | 12.7 | 31.0 | 16.85 | 3.5 |
| BRA9 | Juruaia | 24.2 | 13.3 | 18.3 | 40253 | 34681 | 0.75 | 12.3 | 30.3 | 16.37 | 3.1 |
| BRA10 | Cabo Verde | 24.3 | 13.2 | 18.2 | 40217 | 35262 | 0.56 | 12.8 | 32.6 | 16.61 | 3.2 |

**Table S2.** Brazilian samples mean values for environmental parameters related to the specific cultivation sites during maturation period (April to June 2021). Elevation (elev, above sea level), relative humidity (Rh), cloud cover (Cc), soil temperature at 7, 28, 100, 255 cm depth (sT7, sT28, sT100, sT255), and soil moisture at 7, 28, 100, 255 cm depth (sm7, sm28, sm100, sm255). Data were sourced from Visual Crossing Weather 2020-2021 database (Visual Crossing Corporation).

| Sample | Location | elev (m a.s.l.) | Rh (%) | Cc (%) | sT7 (°C) | sT28 (°C) | sT100 (°C) | sT255 (°C) | sm7 (m³/m³) | sm28 (m³/m³) | sm100 (m³/m³) | sm255 (m³/m³) |
|---|---|---|---|---|---|---|---|---|---|---|---|---|
| BRA1 | Divisa Nova | 894 | 72.53 | 25.19 | 18.4 | 18.7 | 19.7 | 21.1 | 0.258 | 0.275 | 0.312 | 0.355 |
| BRA2 | Capelinha | 928 | 73.86 | 33.95 | 19.9 | 20.2 | 20.9 | 21.9 | 0.312 | 0.351 | 0.412 | 0.332 |
| BRA3 | Patos De Minas | 841 | 60.15 | 24.98 | 22.9 | 23.0 | 23.4 | 23.8 | 0.212 | 0.300 | 0.355 | 0.374 |
| BRA4 | Araponga | 958 | 77.96 | 34.49 | 18.9 | 19.2 | 20.0 | 21.2 | 0.386 | 0.379 | 0.396 | 0.471 |
| BRA5 | São Gotardo | 1061 | 64.02 | 27.09 | 20.2 | 20.4 | 20.9 | 21.6 | 0.195 | 0.219 | 0.267 | 0.373 |
| BRA6 | Campestre | 1070 | 70.02 | 23.13 | 18.3 | 18.6 | 19.5 | 20.9 | 0.312 | 0.359 | 0.412 | 0.444 |
| BRA7 | Tapira | 1088 | 66.43 | 24.68 | 19.5 | 19.8 | 20.5 | 21.5 | 0.329 | 0.351 | 0.390 | 0.427 |
| BRA8 | Monte Santo De Minas | 884 | 64.97 | 21.53 | 19.8 | 20.1 | 21.0 | 22.3 | 0.306 | 0.333 | 0.377 | 0.402 |
| BRA9 | Juruaia | 874 | 71.82 | 26.33 | 18.8 | 19.1 | 20.1 | 21.6 | 0.364 | 0.374 | 0.411 | 0.465 |
| BRA10 | Cabo Verde | 894 | 72.53 | 25.19 | 18.4 | 18.7 | 19.7 | 21.1 | 0.258 | 0.275 | 0.312 | 0.355 |

**Table S3.** Free amino acid content (µg/gFW) in green coffee bean samples from Brazil (BRA1-10), Rwanda (RWA1-4), India (IND1-4), Ethiopia (ETH1-4), Guatemala (GUA1-4), and Honduras (HON1-4). Data are the mean (n = 3) ± SD. Lowercase letters (a, b and c) represent statistically significant differences among samples determined by ANOVA test followed by Tukey post-hoc test ($p < 0.05$) or Kruskal-Wallis test followed by Dunn test ($p < 0.05$).

| | **POLAR** | | | **NON-POLAR** | | | | | | | **ACIDIC** | | **BASIC** | | | **TOTAL** |
|---|---|---|---|---|---|---|---|---|---|---|---|---|---|---|---|---|
| **Sample** | **ser** | **thr** | **tyr** | **gly** | **ala** | **pro** | **val** | **met** | **ile** | **leu** | **phe** | **asp** | **glu** | **hys** | **arg** | **lys** | |
| BRA1 | 113.2$^{ab}$ ± 5.2 | 8.5$^{ab}$ ±1.8 | 7.7$^{ab}$ ±0.5 | 3.6$^{abc}$ ±0.6 | 32.3$^{ab}$ ±1.7 | 15.5$^{a}$ ±1.0 | 13.7$^{abc}$ ±0.5 | 1.6$^{c}$ ±0.1 | 8.1$^{ab}$ ±0.4 | 9.8$^{ab}$ ±0.6 | 53.0$^{ab}$ ±3.1 | 92.5$^{ab}$ ±5.5 | 218.1$^{ab}$ ±12.1 | 18.3$^{ab}$ ±1.5 | 26.3$^{abc}$ ±1.6 | 9.3$^{ab}$ ±0.4 | 631.5$^{abc}$ ±36.5 |
| BRA2 | 128.5$^{ab}$ ± 3.2 | 8.2$^{ab}$ ±0.2 | 6.8$^{a}$ ±0.3 | 1.8$^{c}$ ±0.5 | 32.9$^{ab}$ ±1.7 | 16.9$^{ab}$ ±1.1 | 12.8$^{abc}$ ±0.5 | 1.7$^{bc}$ ±0.1 | 6.5$^{ab}$ ±1.2 | 8.2$^{ab}$ ±0.7 | 47.5$^{ab}$ ±1.4 | 111.7$^{ab}$ ±2.7 | 257.7$^{ab}$ ±5.6 | 14.7$^{abc}$ ±0.9 | 29.0$^{abc}$ ±2.0 | 10.0$^{ab}$ ±0.5 | 695.0$^{abc}$ ±22.5 |
| BRA3 | 120.8$^{ab}$ ± 10.2 | 8.6$^{ab}$ ±1.4 | 6.8$^{ab}$ ±0.9 | 3.1$^{abc}$ ±1.4 | 34.7$^{ab}$ ±3.3 | 17.1$^{ab}$ ±2.4 | 14.0$^{abc}$ ±1.2 | 1.9$^{abc}$ ±0.1 | 8.3$^{ab}$ ±1.0 | 8.2$^{ab}$ ±0.9 | 47.2$^{ab}$ ±4.3 | 113.9$^{ab}$ ±7.1 | 216.8$^{ab}$ ±13.2 | 15.6$^{abc}$ ±2.2 | 28.4$^{abc}$ ±2.4 | 9.5$^{ab}$ ±0.8 | 654.8$^{abc}$ ±52.8 |
| BRA4 | 121.2$^{ab}$ ±10.2 | 7.1$^{ab}$ ±0.5 | 7.6$^{ab}$ ±0.2 | 2.6$^{abc}$ ±0.7 | 34.6$^{ab}$ ±1.0 | 16.6$^{ab}$ ±1.4 | 14.1$^{abc}$ ±0.3 | 1.9$^{abc}$ ±0.1 | 7.0$^{ab}$ ±1.1 | 9.0$^{ab}$ ±0.4 | 40.1$^{ab}$ ±1.4 | 108.3$^{ab}$ ±3.8 | 243.6$^{ab}$ ±6.2 | 19.0$^{abc}$ ±1.9 | 31.0$^{abc}$ ±1.1 | 10.5$^{ab}$ ±0.3 | 674.4$^{abc}$ ±24.1 |
| BRA5 | 203.4$^{a}$ ±22.5 | 9.6$^{ab}$ ±2.0 | 8.4$^{ab}$ ±1.1 | 2.8$^{abc}$ ±1.2 | 50.1$^{ab}$ ±9.3 | 27.0$^{ab}$ ±1.6 | 22.4$^{ab}$ ±3.1 | 1.9$^{abc}$ ±0.2 | 12.0$^{ab}$ ±2.9 | 12.3$^{ab}$ ±1.6 | 60.0$^{ab}$ ±7.4 | 190.7$^{a}$ ±24.3 | 314.2$^{a}$ ±43.8 | 28.1$^{a}$ ±5.3 | 39.7$^{a}$ ±4.3 | 14.3$^{ab}$ ±1.6 | 997.0$^{a}$ ±132.3 |
| BRA6 | 132.9$^{ab}$ ±3.8 | 7.1$^{ab}$ ±0.5 | 7.9$^{ab}$ ±0.3 | 2.3$^{bc}$ ±0.4 | 37.6$^{ab}$ ±1.3 | 16.1$^{ab}$ ±1.8 | 12.8$^{abc}$ ±0.4 | 1.5$^{c}$ ±0.1 | 5.9$^{a}$ ±0.5 | 8.7$^{ab}$ ±0.3 | 44.8$^{ab}$ ±1.5 | 89.6$^{ab}$ ±2.8 | 280.7$^{a}$ ±8.4 | 19.5$^{abc}$ ±0.7 | 29.7$^{abc}$ ±0.7 | 10.0$^{ab}$ ±0.5 | 707.1$^{abc}$ ±23.9 |
| BRA7 | 154.4$^{ab}$ ±12.8 | 8.8$^{ab}$ ±1.9 | 8.4$^{ab}$ ±0.8 | 2.8$^{abc}$ ±1.4 | 57.7$^{ab}$ ±5.8 | 16.5$^{ab}$ ±0.8 | 18.4$^{abc}$ ±1.4 | 2.2$^{abc}$ ±0.1 | 11.1$^{ab}$ ±0.8 | 11.2$^{ab}$ ±0.9 | 63.3$^{a}$ ±4.9 | 100.4$^{ab}$ ±6.4 | 283.1$^{a}$ ±20.8 | 16.4$^{abc}$ ±5.1 | 36.2$^{ab}$ ± 4.3 | 12.6$^{ab}$ ±1.1 | 803.6$^{ab}$ ±69.2 |
| BRA8 | 145.4$^{ab}$ ±13.1 | 8.9$^{ab}$ ±1.6 | 8.5$^{ab}$ ±0.8 | 3.8$^{abc}$ ±0.7 | 56.1$^{ab}$ ±5.5 | 17.0$^{ab}$ ±2.3 | 16.8$^{abc}$ ±1.6 | 2.0$^{abc}$ ±0.2 | 10.2$^{ab}$ ±1.2 | 12.8$^{ab}$ ±1.1 | 52.8$^{ab}$ ±5.8 | 99.0$^{ab}$ ±7.9 | 231.3$^{ab}$ ±19.9 | 14.1$^{abc}$ ±2.3 | 29.6$^{abc}$ ±4.6 | 11.8$^{ab}$ ±1.3 | 720.2$^{abc}$ ±69.9 |
| BRA9 | 118.4$^{ab}$ ±8.0 | 6.2$^{a}$ ±1.9 | 7.3$^{ab}$ ±0.6 | 3.5$^{abc}$ ±0.4 | 28.8$^{ab}$ ±1.7 | 12.8$^{a}$ ±2.1 | 12.8$^{abc}$ ±0.9 | 2.0$^{abc}$ ±0.2 | 7.0$^{ab}$ ±0.8 | 8.7$^{ab}$ ±0.7 | 46.8$^{ab}$ ±3.5 | 100.5$^{ab}$ ±6.7 | 220.0$^{ab}$ ±15.0 | 17.2$^{abc}$ ±0.9 | 24.8$^{abc}$ ±3.9 | 9.9$^{ab}$ ±0.8 | 626.6$^{abc}$ ±48.2 |
| BRA10 | 146.9$^{ab}$ ±5.0 | 7.4$^{ab}$ ±1.6 | 7.6$^{ab}$ ±0.4 | 4.1$^{abc}$ ±0.3 | 39.1$^{ab}$ ±0.3 | 13.8$^{ab}$ ±0.8 | 13.0$^{abc}$ ±0.4 | 1.6$^{c}$ ±0.1 | 8.0$^{ab}$ ±0.3 | 9.2$^{ab}$ ±0.3 | 55.7$^{ab}$ ±1.7 | 109.6$^{ab}$ ±3.0 | 248.2$^{ab}$ ±8.8 | 22.2$^{abc}$ ±0.4 | 26.7$^{abc}$ ±2.0 | 10.2$^{ab}$ ±0.2 | 723.3$^{abc}$ ±25.6 |
| RWA1 | 75.6$^{ab}$ ±2.0 | 11.2$^{ab}$ ±0.4 | 9.1$^{ab}$ ±0.2 | 5.2$^{a}$ ±0.2 | 39.7$^{ab}$ ±2.4 | 26.9$^{ab}$ ±0.5 | 11.5$^{abc}$ ±0.3 | 2.8$^{ab}$ ±0.1 | 7.2$^{ab}$ ±0.1 | 11.1$^{ab}$ ±0.3 | 39.1$^{ab}$ ±1.1 | 35.0$^{b}$ ±1.2 | 96.5$^{b}$ ±8.1 | 10.5$^{abc}$ ±0.6 | 5.8$^{c}$ ±0.3 | 8.4$^{ab}$ ±0.2 | 395.7$^{c}$ ±17.9 |
| RWA2 | 71.9$^{ab}$ ±2.8 | 11.2$^{ab}$ ±0.5 | 7.8$^{ab}$ ±0.3 | 3.0$^{abc}$ ±0.2 | 25.6$^{ab}$ ±1.7 | 26.6$^{b}$ ±1.6 | 10.2$^{abc}$ ±0.5 | 2.3$^{ab}$ ±0.1 | 7.5$^{ab}$ ±1.4 | 8.3$^{ab}$ ±0.8 | 45.3$^{ab}$ ±2.4 | 55.9$^{ab}$ ±3.0 | 172.3$^{ab}$ ±5.4 | 8.7$^{abc}$ ±0.4 | 9.1$^{abc}$ ±4.4 | 7.9$^{ab}$ ±0.6 | 473.6$^{abc}$ ±26.3 |
| RWA3 | 75.4$^{ab}$ ±3.0 | 12.7$^{ab}$ ±0.6 | 10.3$^{ab}$ ±0.3 | 3.5$^{abc}$ ±0.3 | 30.7$^{ab}$ ±1.5 | 30.6$^{b}$ ±1.4 | 10.7$^{abc}$ ±0.2 | 2.7$^{ab}$ ±0.1 | 8.0$^{ab}$ ±0.3 | 9.0$^{ab}$ ±0.5 | 54.0$^{ab}$ ±1.4 | 67.5$^{ab}$ ±2.7 | 184.3$^{ab}$ ±7.4 | 12.4$^{abc}$ ±0.4 | 11.4$^{abc}$ ±0.4 | 8.2$^{b}$ ±0.3 | 531.5$^{abc}$ ±20.8 |
| RWA4 | 88.6$^{ab}$ ±6.9 | 12.9$^{ab}$ ±0.2 | 7.5$^{ab}$ ±0.7 | 3.2$^{abc}$ ±0.1 | 29.0$^{ab}$ ±1.3 | 16.9$^{ab}$ ±1.7 | 9.5$^{c}$ ±0.4 | 1.8$^{bc}$ ±0.3 | 6.9$^{ab}$ ±0.4 | 7.7$^{b}$ ±0.8 | 40.5$^{ab}$ ±2.7 | 54.1$^{ab}$ ±2.1 | 176.6$^{ab}$ ±7.4 | 8.7$^{abc}$ ±0.5 | 10.5$^{abc}$ ±0.9 | 7.6$^{ab}$ ±0.3 | 481.8$^{bc}$ ±26.7 |
| IND1 | 122.1$^{b}$ ±3.4 | 11.9$^{ab}$ ±0.8 | 9.3$^{ab}$ ±0.4 | 4.1$^{abc}$ ±0.8 | 33.1$^{b}$ ±1.0 | 30.9$^{ab}$ ±1.7 | 14.6$^{abc}$ ±0.7 | 2.7$^{abc}$ ±0.1 | 9.3$^{ab}$ ±0.4 | 9.5$^{ab}$± 0.5 | 33.8$^{ab}$ ±1.3 | 96.5$^{ab}$ ±3.4 | 194.0$^{ab}$ ±7.3 | 8.2$^{abc}$ ±0.4 | 13.3$^{bc}$ ±1.4 | 9.3$^{ab}$ ±0.9 | 602.7$^{bc}$ ±24.4 |
| IND2 | 107.4$^{ab}$ ±26.2 | 10.6$^{ab}$ ±2.6 | 7.5$^{ab}$ ±1.6 | 2.9$^{abc}$ ±0.6 | 26.7$^{ab}$ ±5.0 | 23.5$^{ab}$ ±6.3 | 13.1$^{bc}$ ±3.3 | 2.0$^{abc}$ ±0.5 | 8.7$^{ab}$ ±1.9 | 9.5$^{ab}$ ±1.8 | 32.8$^{ab}$ ±8.3 | 73.4$^{ab}$ ±15.7 | 175.2$^{ab}$ ±40.1 | 7.9$^{abc}$ ±2.3 | 10.2$^{abc}$ ±3.8 | 10.0$^{ab}$ ±5.1 | 521.4$^{abc}$ ±125.3 |
| IND3 | 116.6$^{ab}$ ±6.2 | 14.3$^{b}$ ±8.2 | 9.3$^{ab}$ ±0.2 | 3.8$^{abc}$ ±0.1 | 34.3$^{ab}$ ±1.4 | 19.7$^{ab}$ ±0.6 | 14.4$^{abc}$ ±0.4 | 2.4$^{abc}$ ±0.1 | 10.1$^{ab}$ ±0.5 | 10.1$^{ab}$ ±0.4 | 32.0$^{ab}$ ±2.6 | 95.7$^{ab}$ ±6.2 | 210.2$^{ab}$ ±13.4 | 9.3$^{abc}$ ±0.3 | 13.3$^{abc}$ ±5.6 | 10.2$^{ab}$ ±0.3 | 605.5$^{abc}$ ±39.1 |
| IND4 | 138.8$^{ab}$ ±7.8 | 8.2$^{ab}$ ±0.3 | 11.0$^{ab}$ ±0.3 | 3.9$^{abc}$ ±0.4 | 42.0$^{ab}$ ±2.3 | 18.4$^{ab}$ ±01.5 | 17.2$^{abc}$ ±0.6 | 2.2$^{abc}$ ±0.2 | 11.2$^{ab}$ ±0.7 | 13.2$^{ab}$ ±0.3 | 32.4$^{ab}$ ±1.4 | 90.9$^{ab}$ ±4.8 | 231.0$^{ab}$ ±11.8 | 8.2$^{abc}$ ±1.4 | 27.6$^{abc}$ ±1.4 | 12.3$^{ab}$ ±0.4 | 668.6$^{abc}$ ±35.5 |
| ETH1 | 68.4$^{b}$ ±4.3 | 11.7$^{ab}$ ±1.0 | 9.7$^{ab}$ ±0.9 | 4.2$^{abc}$ ±0.9 | 35.1$^{ab}$ ±0.8 | 15.8$^{ab}$ ±0.6 | 11.9± 1.0$^{abc}$ | 1.8$^{bc}$ ±0.3 | 8.6$^{ab}$ ±0.5 | 10.0$^{ab}$ ±1.3 | 29.3$^{ab}$ ±2.4 | 43.2$^{b}$ ±5.0 | 184.9$^{ab}$ ±18.7 | 7.6$^{abc}$ ±0.3 | 14.3$^{abc}$ ±14.3 | 9.0$^{ab}$ ±0.3 | 465.7$^{bc}$ ±42.6 |
| ETH2 | 78.3$^{ab}$ ±5.8 | 14.6$^{b}$ ±1.3 | 10.5$^{ab}$ ±1.2 | 4.1$^{abc}$ ±0.4 | 43.8$^{ab}$ ±5.6 | 12.6$^{a}$ ±0.9 | 12.5± 1.1$^{abc}$ | 1.8$^{bc}$ ±0.2 | 8.5$^{ab}$ ±0.7 | 11.3$^{ab}$ ±1.0 | 30.9$^{b}$ ±3.1 | 53.0$^{ab}$ ±3.4 | 236.4$^{ab}$ ±10.5 | 10.8$^{abc}$ ±0.9 | 23.4$^{abc}$ ±0.9 | 10.6$^{ab}$ ±0.9 | 563.3$^{abc}$ ±37.9 |
| ETH3 | 85.5$^{ab}$ ±6.4 | 9.2$^{ab}$ ±0.7 | 11.6$^{ab}$ ±1.1 | 4.1$^{abc}$ ±0.9 | 58.2$^{ab}$ ±5.2 | 15.2$^{ab}$ ±0.5 | 12.9± 1.1$^{abc}$ | 2.0$^{abc}$ ±0.1 | 8.6$^{ab}$ ±0.6 | 9.7± 0.9$^{ab}$ | 34.3$^{ab}$ ±3.2 | 65.2$^{ab}$ ±5.5 | 195.5$^{ab}$ ±14.6 | 6.4$^{c}$ ±1.1 | 21.2$^{abc}$ ±1.9 | 9.8$^{ab}$ ±0.9 | 549.4$^{abc}$ ±44.6 |
| ETH4 | 98.3$^{ab}$ ±13.7 | 8.6$^{ab}$ ±0.8 | 13.8$^{ab}$ ±2.4 | 4.9$^{ab}$ ±2.8 | 50.8$^{ab}$ ±6.4 | 16.1$^{ab}$ ±2.9 | 15.8± 1.8$^{abc}$ | 2.2$^{abc}$ ±0.2 | 10.0$^{ab}$ ±2.1 | 13.2$_{ab}$ ±2.9 | 37.6$^{ab}$ ±3.2 | 70.5$^{ab}$ ±7.3 | 235.4$^{ab}$ ±15.4 | 10.6$^{abc}$ ±0.9 | 30.1$^{abc}$ ±1.8 | 13.5$^{ab}$ ±1.8 | 631.3$^{abc}$ ±66.3 |
| GUA1 | 145.7$^{ab}$ ±4.7 | 7.5$^{ab}$ ±1.1 | 11.9$^{ab}$ ±0.4 | 3.4$^{abc}$ ±0.4 | 44.8$^{ab}$ ±1.4 | 22.6$^{ab}$ ±0.7 | 17.6± 3.8$^{abc}$ | 2.5$^{ab}$ ±0.1 | 12.2$^{ab}$ ±0.3 | 15.0$^{ab}$ ±0.4 | 42.6$^{ab}$ ±2.0 | 99.5$^{ab}$ ±3.6 | 231.5$^{ab}$ ±8.7 | 14.8$^{abc}$ ±0.7 | 24.7$^{abc}$ ±0.9 | 13.9$^{ab}$ ±0.3 | 710.0$^{abc}$ ±29.4 |
| GUA2 | 140.2$^{ab}$ ±13.8 | 7.5$^{ab}$ ±0.6 | 10.9$^{ab}$ ±0.8 | 3.4$^{abc}$ ±0.3 | 47.4$^{ab}$ ±3.9 | 15.6$^{ab}$ ±1.6 | 19.0± 1.5$^{abc}$ | 2.1$^{abc}$ ±0.2 | 12.1$^{ab}$ ±0.9 | 15.0$^{ab}$ ±1.2 | 40.9$^{ab}$ ±3.3 | 102.8$^{ab}$ ±9.1 | 225.4$^{ab}$ ±16.8 | 14.8$^{abc}$ ±1.4 | 29.3$^{abc}$ ±2.4 | 15.3$^{ab}$ ±1.2 | 701.6$^{abc}$ ±59.1 |
| GUA3 | 179.6$^{a}$ ±11.8 | 7.7$^{ab}$ ±1.1 | 14.3$^{b}$ ±0.9 | 5.0$^{ab}$ ±1.6 | 57.0$^{ab}$ ±4.8 | 21.8$^{ab}$ ±2.7 | 25.2± 1.9$^{a}$ | 3.0$^{a}$ ±0.2 | 16.7$^{b}$ ±1.7 | 19.2$^{ab}$ ±1.5 | 42.7$^{ab}$ ±3.1 | 117.4$^{ab}$ ±7.5 | 211.9$^{ab}$ ±13.8 | 14.6$^{abc}$ ±2.3 | 28.6$^{abc}$ ±1.9 | 16.6$^{a}$ ±1.2 | 781.2$^{abc}$ ±58.3 |
| GUA4 | 148.3$^{ab}$ ±18.3 | 8.1$^{ab}$ ±1.2 | 9.9$^{ab}$ ±0.9 | 4.2$^{abc}$ ±0.6 | 39.3$^{ab}$ ±4.7 | 19.9$^{ab}$ ±1.6 | 17.3± 2.0$^{abc}$ | 2.1$^{abc}$ ±0.3 | 10.8$^{ab}$ ±1.3 | 11.9$^{a}$ ±1.5 | 35.6$^{ab}$ ±4.5 | 109.1$^{ab}$ ±13.1 | 193.7$^{ab}$ ±22.0 | 12.0$^{abc}$ ±1.7 | 28$^{abc}$ ± 5.1 | 12.3$^{ab}$ ±1.5 | 662.4$^{abc}$ ±80.4 |
| HON1 | 144.1$^{ab}$ ±3.4 | 10.4$^{ab}$ ±0.4 | 10.9$^{ab}$ ±0.3 | 4.5$^{abc}$ ±0.2 | 45.5$^{ab}$ ±4.8 | 17.9$^{ab}$ ±0.5 | 18.0± 0.6$^{abc}$ | 2.2$^{abc}$ ±0.1 | 11.8$^{ab}$ ±0.4 | 14.6$^{ab}$ ±0.5 | 37.9$^{ab}$ ±1.0 | 91.5$^{ab}$ ±9.3 | 185.0$^{ab}$ ±21.0 | 11.7$^{abc}$ ±0.7 | 26.5$^{abc}$ ±0.7 | 12.6$^{ab}$ ±0.8 | 645.3$^{abc}$ ±44.8 |
| HON2 | 143.5$^{ab}$ ±8.0 | 8.7$^{ab}$ ±0.9 | 13.0$^{ab}$ ±0.6 | 5.2$^{ab}$ ±0.5 | 61.2$^{a}$ ±3.5 | 20.6$^{ab}$ ±0.8 | 22.2± 1.0$^{ab}$ | 3.0$^{a}$ ±0.1 | 15.1$^{b}$ ±0.7 | 17.9$^{ab}$ ±0.7 | 41.2$^{ab}$ ± 1.8 | 102.5$^{ab}$ ±3.8 | 202.1$^{ab}$ ±11.0 | 12.9$^{abc}$ ±1.3 | 27.3$^{abc}$ ±1.2 | 16.0$^{a}$ ±0.6 | 712.4$^{abc}$ ±36.7 |
| HON3 | 147.2$^{ab}$ ±12.7 | 7.6$^{ab}$ ±1.0 | 11.4$^{ab}$ ±1.0 | 5.7$^{a}$ ±0.4 | 51.3$^{ab}$ ±9.6 | 21.6$^{ab}$ ±2.7 | 18.7± 1.8$^{abc}$ | 2.2$^{abc}$ ±0.2 | 12.4$^{ab}$ ±0.8 | 14.7$^{ab}$ ±1.3 | 35.9$^{ab}$ ± 3.5 | 99.8$^{ab}$ ±10.4 | 193.4$^{ab}$ ±21.8 | 8.0$^{abc}$ ±0.4 | 25.3$^{abc}$ ±1.3 | 13.7$^{ab}$ ±1.3 | 668.8$^{abc}$ ±70.3 |
| HON4 | 125.3$^{ab}$ ±23.7 | 6.3$^{a}$ ±1.1 | 11.8$^{ab}$ ±2.7 | 5.2$^{ab}$ ±1.3 | 56.5$^{ab}$ ±4.8 | 21.3$^{ab}$ ±4.5 | 18.6± 3.7$^{abc}$ | 2.3$^{abc}$ ±0.4 | 12.4$^{ab}$ ±2.6 | 14.6$^{ab}$ ±2.8 | 35.0$^{ab}$ ±6.7 | 91.7$^{ab}$ ±17.5 | 196.2$^{ab}$ ±37.9 | 9.1$^{abc}$ ±1.8 | 24.0$^{abc}$ ±3.6 | 12.8$^{ab}$ ±2.2 | 643.1$^{abc}$ ±117.1 |

**Figure S1.** Principal Component Analysis (PCA) score plots for specific compound subsets: (**a**), 3-, 5-, 4-caffeoylquinic acids; (**b**) caffeine and trigonelline; (**c**) free amino acids (ser, thr, tyr, gly, ala, pro, val, met, ile, leu, phe, asp, glu, hys, arg, lys). Each point represents sample replicates and the ellipses indicate 95% confidence intervals around the mean data of each country. The analyses were conducted using R program (R Core Team version 4.1.2, 2022). The *prcomp()* function was used for principal component computation. The *factoextra* package was employed for plot visualization.

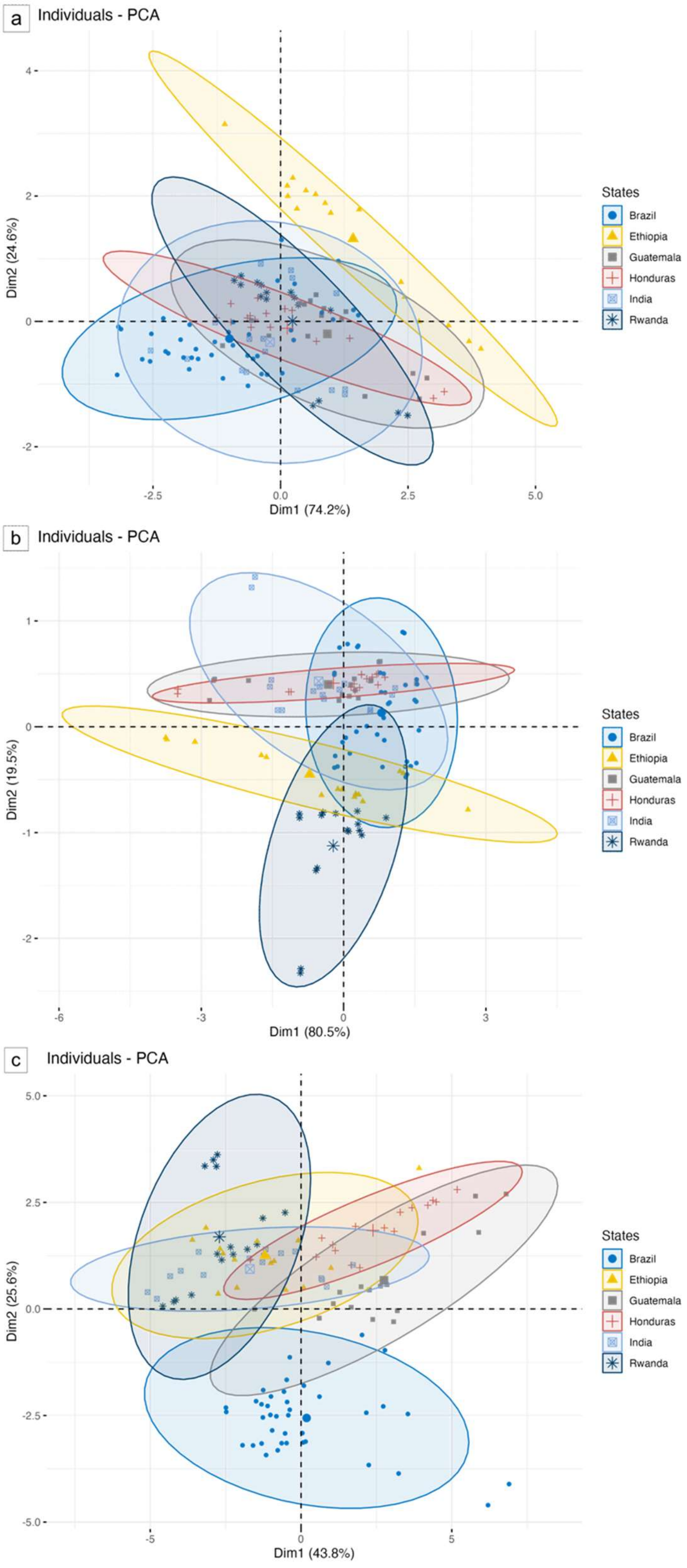

**Table S4.** Matrix including Pearson correlation coefficients between phytochemical data of green coffee beans from Brazil (BRA1-10) and meteorological parameters of cultivation sites (Table S1). TOTP, total polyphenols; 3-CQA, 5-CQA, 4-CQA: 3-, 5-, 4-caffeoylquinic acids; CAF, caffeine; TRI, trigonelline; SUC, sucrose; POLAR, NON-POLAR, ACIDIC, BASIC: free polar, non-polar, acidic and basic amino acids; PUT, putrescine; SPD, spermidine; SPM, spermine; TRP, tryptamine; Tmax, Tmin, Tmean: maximum, minimum and mean temperatures; day_dur, daylight duration; sun_dur, sunshine duration; precip, rainfall; wnd_spd, average wind speed; wnd_gst, maximum wind gusts; sw_rad, shortwave radiation; evap, evapotranspiration. To indicate the significance of correlations, asterisks were assigned: $p < 0.001$ (highly significant, ***), $p < 0.01$ (**), $p < 0.05$ (*), $p \geq 0.05$ (not significant, no asterisk).

Pearson Correlation Matrix

| | TOTP | 3-CQA | 5-CQA | 4-CQA | CAF | TRI | SUC | POLAR | NON-POLAR | ACIDIC | BASIC | PUT | SPD | SPM | TRP | Tmax | Tmin | Tmean | day_dur | sun_dur | precip | wnd_spd | wnd_gst | sw_rad | evap |
|---|---|---|---|---|---|---|---|---|---|---|---|---|---|---|---|---|---|---|---|---|---|---|---|---|---|
| TOTP | 1NA | -0.102 | 0.006 | -0.028 | -0.128 | -0.076 | 0.176 | 0.06 | -0.056 | 0.113 | 0 | 0.049 | 0.117 | 0.105 | -0.068 | 0.052 | -0.08 | 0.013 | -0.166 | -0.002 | -0.111 | -0.018 | -0.227 | 0.089 | 0.112 |
| 3-CQA | -0.102 | 1NA | 0.494** | 0.953*** | 0.321* | 0.536*** | 0.212 | 0.373* | 0.48** | 0.144 | 0.146 | 0.333* | 0.272 | 0.288 | 0.47** | 0.611*** | 0.266 | 0.481** | 0.148 | -0.079 | -0.002 | -0.301 | -0.388* | 0.48** | 0.545*** |
| 5-CQA | 0.006 | 0.494** | 1NA | 0.604*** | 0.835*** | 0.371* | -0.03 | 0.439** | 0.436** | 0.362* | 0.374* | -0.208 | -0.333* | -0.294 | 0.286 | -0.085 | -0.234 | -0.161 | -0.128 | 0.014 | 0.097 | 0.379* | 0.048 | 0.203 | 0.174 |
| 4-CQA | -0.028 | 0.953*** | 0.604*** | 1NA | 0.427** | 0.559*** | 0.211 | 0.386* | 0.495** | 0.16 | 0.147 | 0.277 | 0.205 | 0.209 | 0.43** | 0.473** | 0.136 | 0.336* | 0.103 | -0.112 | 0.029 | -0.157 | -0.329* | 0.409** | 0.462** |
| CAF | -0.128 | 0.321* | 0.835*** | 0.427** | 1NA | 0.37* | -0.101 | 0.379* | 0.422** | 0.34* | 0.4* | -0.225 | -0.363* | -0.298 | 0.072 | -0.258 | -0.318* | -0.318* | -0.191 | -0.025 | 0.246 | 0.487** | 0.263 | 0.026 | -0.03 |
| TRI | -0.076 | 0.536*** | 0.371* | 0.559*** | 0.37* | 1NA | 0.201 | -0.074 | 0.201 | -0.34* | -0.266 | -0.183 | -0.071 | -0.072 | 0.058 | 0.285 | -0.343* | -0.032 | -0.325* | -0.404** | -0.073 | -0.115 | -0.143 | 0.088 | 0.14 |
| SUC | 0.176 | 0.212 | -0.03 | 0.211 | -0.101 | 0.201 | 1NA | -0.07 | -0.073 | -0.059 | -0.152 | 0.641*** | 0.672*** | 0.623*** | -0.192 | 0.491** | -0.124 | 0.24 | 0.224 | -0.209 | -0.743*** | -0.221 | -0.464** | 0.374* | 0.375* |
| POLAR | 0.06 | 0.373* | 0.439** | 0.386* | 0.379* | -0.074 | -0.07 | 1NA | 0.874*** | 0.918*** | 0.908*** | 0.258 | -0.01 | 0.047 | 0.572*** | 0.112 | 0.218 | 0.189 | 0.175 | 0.413** | 0.035 | 0.283 | 0.087 | 0.462** | 0.413** |
| NON-POLAR | -0.056 | 0.48** | 0.436** | 0.495** | 0.422** | 0.201 | -0.073 | 0.874*** | 1NA | 0.682*** | 0.76*** | 0.176 | -0.071 | -0.083 | 0.445** | 0.249 | 0.245 | 0.272 | 0.094 | 0.492** | 0.117 | 0.373* | 0.125 | 0.585*** | 0.526*** |
| ACIDIC | 0.113 | 0.144 | 0.362* | 0.16 | 0.34* | -0.34* | -0.059 | 0.918*** | 0.682*** | 1NA | 0.925*** | 0.252 | -0.034 | 0.049 | 0.409** | -0.055 | 0.205 | 0.095 | 0.322* | 0.386* | -0.069 | 0.268 | 0.083 | 0.318* | 0.26 |
| BASIC | 0 | 0.146 | 0.374* | 0.147 | 0.4* | -0.266 | -0.152 | 0.908*** | 0.76*** | 0.925*** | 1NA | 0.22 | -0.062 | -0.006 | 0.381* | -0.077 | 0.181 | 0.06 | 0.138 | 0.476** | 0.107 | 0.404** | 0.258 | 0.326* | 0.242 |
| PUT | 0.049 | 0.333* | -0.208 | 0.277 | -0.225 | -0.183 | 0.641*** | 0.258 | 0.176 | 0.252 | 0.22 | 1NA | 0.91*** | 0.88*** | 0.097 | 0.567*** | 0.433** | 0.564*** | 0.456** | 0.207 | -0.373* | -0.254 | -0.366* | 0.509*** | 0.502*** |
| SPD | 0.117 | 0.272 | -0.333* | 0.205 | -0.363* | -0.071 | 0.672*** | -0.01 | -0.071 | -0.034 | -0.062 | 0.91*** | 1NA | 0.941*** | -0.02 | 0.569*** | 0.328* | 0.506*** | 0.301 | 0.028 | -0.354* | -0.384* | -0.426** | 0.369* | 0.393* |
| SPM | 0.105 | 0.288 | -0.294 | 0.209 | -0.298 | -0.072 | 0.623*** | 0.047 | -0.083 | 0.049 | -0.006 | 0.88*** | 0.941*** | 1NA | 0.054 | 0.502*** | 0.292 | 0.448** | 0.317* | -0.047 | -0.352* | -0.427** | -0.417** | 0.291 | 0.324* |
| TRP | -0.068 | 0.47** | 0.286 | 0.43** | 0.072 | 0.058 | -0.192 | 0.572*** | 0.445** | 0.409** | 0.381* | 0.097 | -0.02 | 0.054 | 1NA | 0.187 | 0.21 | 0.213 | -0.038 | 0.195 | 0.171 | -0.025 | 0.024 | 0.282 | 0.298 |
| Tmax | 0.052 | 0.611*** | -0.085 | 0.473** | -0.258 | 0.285 | 0.491** | 0.112 | 0.249 | -0.055 | -0.077 | 0.567*** | 0.569*** | 0.502*** | 0.187 | 1NA | 0.594*** | 0.903*** | 0.389* | 0.183 | -0.446** | -0.552*** | -0.743*** | 0.795*** | 0.872*** |
| Tmin | -0.08 | 0.266 | -0.234 | 0.136 | -0.318* | -0.343* | -0.124 | 0.218 | 0.245 | 0.205 | 0.181 | 0.433** | 0.328* | 0.292 | 0.21 | 0.594*** | 1NA | 0.878*** | 0.672*** | 0.634*** | -0.026 | -0.337* | -0.419** | 0.594*** | 0.632*** |
| Tmean | 0.013 | 0.481** | -0.161 | 0.336* | -0.318* | -0.032 | 0.24 | 0.189 | 0.272 | 0.095 | 0.06 | 0.564*** | 0.506*** | 0.448** | 0.213 | 0.903*** | 0.878*** | 1NA | 0.604*** | 0.456** | -0.312* | -0.496** | -0.682*** | 0.8*** | 0.864*** |
| day_dur | -0.166 | 0.148 | -0.128 | 0.103 | -0.191 | -0.325* | 0.224 | 0.175 | 0.094 | 0.322* | 0.138 | 0.456** | 0.301 | 0.317* | -0.038 | 0.389* | 0.672*** | 0.604*** | 1NA | 0.276 | -0.532*** | -0.372* | -0.542*** | 0.391* | 0.427** |
| sun_dur | -0.002 | -0.079 | 0.014 | -0.112 | -0.025 | -0.404** | -0.209 | 0.413** | 0.492** | 0.386* | 0.476** | 0.207 | 0.028 | -0.047 | 0.195 | 0.183 | 0.634*** | 0.456** | 0.276 | 1NA | 0.121 | 0.464** | 0.177 | 0.629*** | 0.525*** |
| precip | -0.111 | -0.002 | 0.097 | 0.029 | 0.246 | -0.073 | -0.743*** | 0.035 | 0.117 | -0.069 | 0.107 | -0.373* | -0.354* | -0.352* | 0.171 | -0.446** | -0.026 | -0.312* | -0.532*** | 0.121 | 1NA | 0.363* | 0.615*** | -0.371* | -0.401* |
| wnd_spd | -0.018 | -0.301 | 0.379* | -0.157 | 0.487** | -0.115 | -0.221 | 0.283 | 0.373* | 0.268 | 0.404** | -0.254 | -0.384* | -0.427** | -0.025 | -0.552*** | -0.337* | -0.496** | -0.372* | 0.464** | 0.363* | 1NA | 0.758*** | -0.002 | -0.171 |
| wnd_gst | -0.227 | -0.388* | 0.048 | -0.329* | 0.263 | -0.143 | -0.464** | 0.087 | 0.125 | 0.083 | 0.258 | -0.366* | -0.426** | -0.417** | 0.024 | -0.743*** | -0.419** | -0.682*** | -0.542*** | 0.177 | 0.615*** | 0.758*** | 1NA | -0.452** | -0.594*** |
| sw_rad | 0.089 | 0.48** | 0.203 | 0.409** | 0.026 | 0.088 | 0.374* | 0.462** | 0.585*** | 0.318* | 0.326* | 0.509*** | 0.369* | 0.291 | 0.282 | 0.795*** | 0.594*** | 0.8*** | 0.391* | 0.629*** | -0.371* | -0.002 | -0.452** | 1NA | 0.982*** |
| evap | 0.112 | 0.545*** | 0.174 | 0.462** | -0.03 | 0.14 | 0.375* | 0.413** | 0.526*** | 0.26 | 0.242 | 0.502*** | 0.393* | 0.324* | 0.298 | 0.872*** | 0.632*** | 0.864*** | 0.427** | 0.525*** | -0.401* | -0.171 | -0.594*** | 0.982*** | 1NA |

**Table S5.** Matrix including Pearson correlation coefficients between phytochemical data of green coffee beans from Brazil (BRA1-10) and environmental parameters of cultivation sites (Table S2). TOTP, total polyphenols; 3-CQA, 5-CQA, 4-CQA: 3-, 5-, 4-caffeoylquinic acids; CAF, caffeine; TRI, trigonelline; SUC, sucrose; POLAR, NON-POLAR, ACIDIC, BASIC: free polar, non-polar, acidic and basic amino acids; PUT, putrescine; SPD, spermidine; SPM, spermine; TRP, tryptamine; elev, altitude above sea level; Rh, relative humidity; Cc, cloud cover; sT7, sT28, sT100, sT255: soil temperature at 7, 28, 100, 255 cm depth; sm7, sm28, sm100, sm255: soil moisture at 7, 28, 100, 255 cm depth. To indicate the significance of correlations, asterisks were assigned: $p < 0.001$ (highly significant, ***), $p < 0.01$ (**), $p < 0.05$ (*), $p \geq 0.05$ (not significant, no asterisk).

Pearson Correlation Matrix

| | TOTP | 3-CQA | 5-CQA | 4-CQA | CAF | TRI | SUC | POLAR | NON-POLAR | ACIDIC | BASIC | PUT | SPD | SPM | TRP | elev | Rh | Cc | sT7 | sT28 | sT100 | sT255 | sm7 | sm28 | sm100 | sm255 |
|---|---|---|---|---|---|---|---|---|---|---|---|---|---|---|---|---|---|---|---|---|---|---|---|---|---|---|
| TOTP | 1NA | -0.102 | 0.006 | -0.028 | -0.128 | -0.076 | 0.176 | 0.06 | -0.056 | 0.113 | 0 | 0.049 | 0.117 | 0.105 | -0.068 | 0.029 | -0.207 | -0.303 | 0.073 | 0.073 | 0.085 | 0.098 | -0.083 | 0.073 | 0.133 | 0.181 |
| X3.CQA | -0.102 | 1NA | 0.494** | 0.953*** | 0.321* | 0.536*** | 0.212 | 0.373* | 0.48** | 0.144 | 0.146 | 0.333* | 0.272 | 0.288 | 0.47** | -0.162 | -0.539*** | -0.383* | 0.295 | 0.29 | 0.293 | 0.335* | -0.328* | -0.368* | -0.325* | -0.24 |
| X5.CQA | 0.006 | 0.494** | 1NA | 0.604*** | 0.835*** | 0.371* | -0.03 | 0.439** | 0.436** | 0.362* | 0.374* | -0.208 | -0.333* | -0.294 | 0.286 | 0.583*** | -0.269 | -0.364* | -0.191 | -0.202 | -0.229 | -0.276 | -0.004 | -0.064 | -0.023 | 0.199 |
| X4.CQA | -0.028 | 0.953*** | 0.604*** | 1NA | 0.427** | 0.559*** | 0.211 | 0.386* | 0.495** | 0.16 | 0.147 | 0.277 | 0.205 | 0.209 | 0.43** | -0.018 | -0.483** | -0.398* | 0.189 | 0.185 | 0.188 | 0.23 | -0.159 | -0.188 | -0.139 | -0.11 |
| CAF | -0.128 | 0.321* | 0.835*** | 0.427** | 1NA | 0.37* | -0.101 | 0.379* | 0.422** | 0.34* | 0.4* | -0.225 | -0.363* | -0.298 | 0.072 | 0.6*** | -0.046 | -0.197 | -0.344* | -0.354* | -0.382* | -0.431** | 0.199 | 0.06 | 0.042 | 0.33* |
| TRI | -0.076 | 0.536*** | 0.371* | 0.559*** | 0.37* | 1NA | 0.201 | -0.074 | 0.201 | -0.34* | -0.266 | -0.183 | -0.071 | -0.072 | 0.058 | -0.246 | -0.159 | -0.584*** | -0.198 | -0.194 | -0.158 | -0.007 | -0.025 | -0.04 | -0.004 | -0.236 |
| SUC | 0.176 | 0.212 | -0.03 | 0.211 | -0.101 | 0.201 | 1NA | -0.07 | -0.073 | -0.059 | -0.152 | 0.641*** | 0.672*** | 0.623*** | -0.192 | -0.094 | -0.28 | -0.252 | 0.225 | 0.217 | 0.215 | 0.228 | -0.378* | -0.207 | -0.101 | -0.389* |
| POLAR | 0.06 | 0.373* | 0.439** | 0.386* | 0.379* | -0.074 | -0.07 | 1NA | 0.874*** | 0.918*** | 0.908*** | 0.258 | -0.01 | 0.047 | 0.572*** | 0.543*** | -0.415** | -0.161 | 0.113 | 0.096 | 0.041 | -0.086 | -0.445** | -0.56*** | -0.534*** | -0.179 |
| NON.POLAR | -0.056 | 0.48** | 0.436** | 0.495** | 0.422** | 0.201 | -0.073 | 0.874*** | 1NA | 0.682*** | 0.76*** | 0.176 | -0.071 | -0.083 | 0.445** | 0.45** | -0.523*** | -0.356* | 0.16 | 0.147 | 0.105 | 0.031 | -0.355* | -0.451** | -0.446** | -0.149 |
| ACIDIC | 0.113 | 0.144 | 0.362* | 0.16 | 0.34* | -0.34* | -0.059 | 0.918*** | 0.682*** | 1NA | 0.925*** | 0.252 | -0.034 | 0.049 | 0.409** | 0.63*** | -0.246 | 0.118 | 0.105 | 0.087 | 0.021 | -0.153 | -0.376* | -0.466** | -0.431** | -0.132 |
| BASIC | 0 | 0.146 | 0.374* | 0.147 | 0.4* | -0.266 | -0.152 | 0.908*** | 0.76*** | 0.925*** | 1NA | 0.22 | -0.062 | -0.006 | 0.381* | 0.614*** | -0.236 | 0.019 | 0.034 | 0.017 | -0.046 | -0.205 | -0.332* | -0.471** | -0.488** | -0.018 |
| PUT | 0.049 | 0.333* | -0.208 | 0.277 | -0.225 | -0.183 | 0.641*** | 0.258 | 0.176 | 0.252 | 0.22 | 1NA | 0.91*** | 0.88*** | 0.097 | -0.136 | -0.391* | 0.029 | 0.535*** | 0.526*** | 0.508*** | 0.456** | -0.428** | -0.338* | -0.305 | -0.274 |
| SPD | 0.117 | 0.272 | -0.333* | 0.205 | -0.363* | -0.071 | 0.672*** | -0.01 | -0.071 | -0.034 | -0.062 | 0.91*** | 1NA | 0.941*** | -0.02 | -0.364* | -0.304 | -0.04 | 0.474** | 0.471** | 0.477** | 0.489** | -0.326* | -0.2 | -0.17 | -0.224 |
| SPM | 0.105 | 0.288 | -0.294 | 0.209 | -0.298 | -0.072 | 0.623*** | 0.047 | -0.083 | 0.049 | -0.006 | 0.88*** | 0.941*** | 1NA | 0.054 | -0.338* | -0.235 | 0.036 | 0.411** | 0.407** | 0.409** | 0.409** | -0.338* | -0.247 | -0.212 | -0.262 |
| TRP | -0.068 | 0.47** | 0.286 | 0.43** | 0.072 | 0.058 | -0.192 | 0.572*** | 0.445** | 0.409** | 0.381* | 0.097 | -0.02 | 0.054 | 1NA | 0.096 | -0.317* | -0.233 | 0.065 | 0.057 | 0.035 | 0.003 | -0.433** | -0.567*** | -0.565*** | -0.248 |
| elev | 0.029 | -0.162 | 0.583*** | -0.018 | 0.6*** | -0.246 | -0.094 | 0.543*** | 0.45** | 0.63*** | 0.614*** | -0.136 | -0.364* | -0.338* | 0.096 | 1NA | -0.044 | -0.01 | -0.257 | -0.272 | -0.337* | -0.503*** | 0.063 | -0.004 | 0.004 | 0.268 |
| Rh | -0.207 | -0.539*** | -0.269 | -0.483** | -0.046 | -0.159 | -0.28 | -0.415** | -0.523*** | -0.246 | -0.236 | -0.391* | -0.304 | -0.235 | -0.317* | -0.044 | 1NA | 0.622*** | -0.745*** | -0.737*** | -0.725*** | -0.696*** | 0.651*** | 0.431** | 0.312* | 0.21 |
| Cc | -0.303 | -0.383* | -0.364* | -0.398* | -0.197 | -0.584*** | -0.252 | -0.161 | -0.356* | 0.118 | 0.019 | 0.029 | -0.04 | 0.036 | -0.233 | -0.01 | 0.622*** | 1NA | -0.027 | -0.023 | -0.041 | -0.131 | 0.35* | 0.262 | 0.215 | -0.008 |
| sT7 | 0.073 | 0.295 | -0.191 | 0.189 | -0.344* | -0.198 | 0.225 | 0.113 | 0.16 | 0.105 | 0.034 | 0.535*** | 0.474** | 0.411** | 0.065 | -0.257 | -0.745*** | -0.027 | 1NA | 1*** | 0.995*** | 0.946*** | -0.511*** | -0.234 | -0.122 | -0.298 |
| sT28 | 0.073 | 0.29 | -0.202 | 0.185 | -0.354* | -0.194 | 0.217 | 0.096 | 0.147 | 0.087 | 0.017 | 0.526*** | 0.471** | 0.407** | 0.057 | -0.272 | -0.737*** | -0.023 | 1*** | 1NA | 0.997*** | 0.953*** | -0.496** | -0.216 | -0.105 | -0.292 |
| sT100 | 0.085 | 0.293 | -0.229 | 0.188 | -0.382* | -0.158 | 0.215 | 0.041 | 0.105 | 0.021 | -0.046 | 0.508*** | 0.477** | 0.409** | 0.035 | -0.337* | -0.725*** | -0.041 | 0.995*** | 0.997*** | 1NA | 0.974*** | -0.465** | -0.177 | -0.067 | -0.279 |
| sT255 | 0.098 | 0.335* | -0.276 | 0.23 | -0.431** | -0.007 | 0.228 | -0.086 | 0.031 | -0.153 | -0.205 | 0.456** | 0.489** | 0.409** | 0.003 | -0.503*** | -0.696*** | -0.131 | 0.946*** | 0.953*** | 0.974*** | 1NA | -0.402* | -0.109 | -0.002 | -0.274 |
| sm7 | -0.083 | -0.328* | -0.004 | -0.159 | 0.199 | -0.025 | -0.378* | -0.445** | -0.355* | -0.376* | -0.332* | -0.428** | -0.326* | -0.338* | -0.433** | 0.063 | 0.651*** | 0.35* | -0.511*** | -0.496** | -0.465** | -0.402* | 1NA | 0.913*** | 0.811*** | 0.714*** |
| sm28 | 0.073 | -0.368* | -0.064 | -0.188 | 0.06 | -0.04 | -0.207 | -0.56*** | -0.451** | -0.466** | -0.471** | -0.338* | -0.2 | -0.247 | -0.567*** | -0.004 | 0.431** | 0.262 | -0.234 | -0.216 | -0.177 | -0.109 | 0.913*** | 1NA | 0.971*** | 0.664*** |
| sm100 | 0.133 | -0.325* | -0.023 | -0.139 | 0.042 | -0.004 | -0.101 | -0.534*** | -0.446** | -0.431** | -0.488** | -0.305 | -0.17 | -0.212 | -0.565*** | 0.004 | 0.312* | 0.215 | -0.122 | -0.105 | -0.067 | -0.002 | 0.811*** | 0.971*** | 1NA | 0.546*** |
| sm255 | 0.181 | -0.24 | 0.199 | -0.11 | 0.33* | -0.236 | -0.389* | -0.179 | -0.149 | -0.132 | -0.018 | -0.274 | -0.224 | -0.262 | -0.248 | 0.268 | 0.21 | -0.008 | -0.298 | -0.292 | -0.279 | -0.274 | 0.714*** | 0.664*** | 0.546*** | 1NA |
